# Insights into the mechanisms of triptolide nephrotoxicity through network pharmacology-based analysis and RNA-seq

**DOI:** 10.3389/fpls.2023.1144583

**Published:** 2023-03-07

**Authors:** Yue-Ming Luo, Shu-Dong Yang, Miao-Yu Wen, Bing Wang, Jia-Hui Liu, Si-Ting Li, Yu-Yan Li, Hong Cheng, Li-Li Zhao, Shun-Min Li, Jian-Jun Jiang

**Affiliations:** ^1^ Department of Nephrology, Shenzhen Traditional Chinese Medicine Hospital, The Fourth Clinical Medical College of Guangzhou University of Chinese Medicine, Shenzhen, China; ^2^ Department of Geriatrics, Shenzhen Traditional Chinese Medicine Hospital, The Fourth Clinical Medical College of Guangzhou University of Chinese Medicine, Shenzhen, China; ^3^ Department of Nephrology, Shenzhen Affiliated Hospital of Nanjing University of Chinese Medicine, Nanjing, China; ^4^ Institute of Basic Research in Clinical Medicine, China Academy of Chinese Medical Sciences, Beijing, China; ^5^ Department of Clinical Laboratory, The Second Affiliated Hospital of Anhui University of Chinese Medicine, Hefei, China; ^6^ Graduate school of Clinical Medicine, Anhui Medical University, Hefei, China

**Keywords:** triptolide, nephrotoxicity, network pharmacology, RNA-seq, noncoding RNA

## Abstract

**Introduction:**

Triptolide (TPL) is a promising plant-derived compound for clinical therapy of multiple human diseases; however, its application was limited considering its toxicity.

**Methods:**

To explore the underlying molecular mechanism of TPL nephrotoxicity, a network pharmacology based approach was utilized to predict candidate targets related with TPL toxicity, followed by deep RNA-seq analysis to characterize the features of three transcriptional elements include protein coding genes (PCGs), long noncoding RNAs (lncRNAs) and circular RNAs (circRNAs) as well as their associations with nephrotoxicity in rats with TPL treatment.

**Results & Discussion:**

Although the deeper mechanisms of TPL nephrotoxcity remain further exploration, our results suggested that c-Jun is a potential target of TPL and Per1 related circadian rhythm signaling is involved in TPL induced renal toxicity.

## Introduction

Over 3000 years of constant practice and optimization for the system of Traditional Chinese Medicine (TCM) have endowed its specific tradition that treasures in both scientific and medical fields (Li et al., 2007). A better understanding of therapeutic mechanisms of herb and herbal formulas from TCMs is of great significance for pharmacological study as they have played vital roles in clinical practice (Zuo et al., 2018). As one of the most renowned traditional Chinese medical herbs, *Tripterygium wilfordii* Hook f. (TWHF) has been applied in the treatment of multiple renal diseases such as membranous nephropathy (MN), nephrotic syndrome (NS) and refractory proteinuria since ancient China. Triptolide (TPL) is a major active component of TWHF as well as a promising compound for cancer therapy (Noel et al., 2019). Increasing evidence suggests that TPL can attenuate the progression of several types of tumor *via* varieties of approaches including target epigenetic networks (Noel et al., 2020), induce cancer cell apoptosis, enhance the effect of radiotherapy, inhibit metastasis and etc (Meng et al., 2014). TPL also shows potential immunosuppressive effect in autoimmune diseases treatment such as rheumatoid arthritis (Fan et al., 2018). However, the clinical application of TPL is restricted due to its hepatic, nephric, heart and gastrointestinal toxicity (Cheng et al., 2021). The cytotoxic activities of TPL include introducing DNA damage and apoptosis, arresting cell cycle (Park and Kim, 2013), autophagy (You et al., 2018), and it involves in the production of reactive oxygen species (ROS), generation and depolarization of mitochondrial membrane potential (MMP) in different cell lines (Zhang et al., 2019).

The advancement of bioinformatics as well as the booming development of compound/drug/diseases databases such as TCMSP (Ru et al., 2014), NIMS (Li et al., 2011) and comCIPHER (Zhao and Li, 2012) have facilitated network pharmacology as a feasible approach to explicate the material composition and molecular mechanism of drugs effectively since it seeks targets by constructing distinct networks and evaluating the molecular connections involved in the process of drug treatment (Li et al., 2019). Network pharmacology has greatly enhanced the investigation of the molecular basis of herbal formula in the past decade (Li and Zhang, 2013). Through network pharmacology, Li et al. revealed the targets and pathways of niacin in the treatment of COVID-19 and colorectal cancer (Li et al., 2021). Niu et al. found that IL6 is potentially regulated by phytochemicals in traditional Chinese medicine for COVID-19 treatment (Niu et al., 2021). On the other hand, RNA-seq has been widely used to affiliate the expression patterns of protein coding gene (PCG), long noncoding RNA (lncRNA) and circular RNA (circRNA). Increasing evidence has shown that lncRNAs and circRNAs are closely related with degenerative diseases (Bhatti et al., 2021), cancers (Anastasiadou et al., 2018) development (Fatica and Bozzoni, 2014; Di Agostino et al., 2020), aging (Jiang et al., 2021; Ge et al., 2022), and they have great potential to be utilized as drug targets in the near future (Matsui and Corey, 2017; He et al., 2021).

Although previous renal metabolic analysis revealed that Toll-like receptor signaling pathway and NF-κB signaling pathway played an important role in TPL-induced nephrotoxicity (Huang et al., 2019), the signatures of transcriptional elements are largely unexplored. In this study, we employed deep RNA-seq in female rat kidneys as well as network pharmacology-based analysis, to elucidate the principles of transcriptomic changes (include protein coding genes, lncRNAs and circRNAs) that associated with TPL and identify candidate targets for a better understanding of TPL renal toxicology.

## Materials and methods

### Animals, pathological measurements and ethic statements

Female Sprague-Dawley (SD) rats, weighing 170-190g, were purchased from Guangdong medical laboratory animal center (Guangzhou, China) and housed in the animal facility of our institute under a pathogen-free condition. Rats were fed in an *ad arbitrium* diet and with free access to water. TPL was purchased from MedChemExpress (New Jersey, USA). Rats were randomly divided into control (Ctrl, n = 3), low dosage of TPL (L-TPL, n = 6) and high dosage (H-TPL, n = 6) groups. The L-TPL and H-TPL rats were separately administrated by oral gavage at a dose of 0.2 mg/kg and 0.4mg/kg for 28 days. Blood samples were collected for testing blood urea nitrogen (BUN) and serum creatinine (Scr) using one-way anova method among three groups. Coronal renal tissue was sectioned for H & E staining following standard protocols. Renal parenchyma was dissected for RNA-seq. The animal protocol of this study was approved by the institutional ethics review board of Shenzhen PKU-HKUST Medical Center (No. 2020252) and the authors declare that all the procedures have carefully followed the animal protocol. This study was in accordance with ARRIVE guidelines (https://arriveguidelines.org).

### RNA isolation and sequencing

Three rats per group were randomly selected from Ctrl and H-TPL groups for RNA-seq. Total RNA was extracted from kidney using Trizol reagent (Invitrogen Cat#15596026) following standard protocols and subjected to the preparation of ribosome depletion RNA sequencing library by illumina platform.

### Data availability

The annotation files of novel lncRNAs and circRNAs and the raw data were submitted to the Genome Sequence Archive in BIG Data Center, (Beijing Institute of Genomics (BIG), Chinese Academy of Sciences (http://bigd.big.ac.cn/gsa) (Chen et al., 2021), under the bioproject PRJCA010363 with accession No. CRA008544.

### Target prediction by network pharmacology-based analysis

A step-wise workflow was utilized to predict candidate targets related with TPL nephrotoxicity. Firstly, TPL related targets (TPL-RT) were collected from TCMSP database (http://tcmspw.com/tcmsp.php) and published studies from PubMed (https://pubmed.ncbi.nlm.nih.gov/) database that related with TPL. Then, we used “nephrotoxicity” as the keyword to acquire the known nephrotoxicity related targets (nephrotoxicity-RT) from GeneCard, OMIN and DRUGBANK databases, respectively. The overlapped targets (OT) between TPL-RT and nephrotoxicity-RT were retained and subjected to STRING database to construct their protein-protein interaction (PPI) networks. Protein pairs with correlation r-value > 0.9 were regarded as high-quality networks (hq-ntw) and were visualized by cytoscape (Shannon et al., 2003). Gene enrichment analysis was employed to classify proteins within hq-ntw.

### Molecular docking analysis

The 2D structure of TPL was downloaded from PubChem database (https://pubchem.ncbi.nlm.nih.gov/), then the structure was subjected to optimization by Chem3D software (https://library.bath.ac.uk/chemistry-software/chem3d). PyMOL (https://pymol.org/2/) was utilized to remove the water molecues and small ligands from the protein structures of targets downloaded from PDB database (http://www.rcsb.org/) for subsequent step. The molecular docking was finally performed and visualized using the hydrogen bonded protein structure and optimized TPL structure *via* AutoDockTools software (https://www.scripps.edu/sanner/software/adt/Tutorial/index.html).

### Western-blot

Western-blot assay was performed as we have previously described (Jiang et al., 2021), blots were cut according to the sizes of target proteins prior to hybridisation with antibodies during blotting and exposed by Bio-rad imaging system. Antibody information see **Supplementary Table 1**.

### Novel lncRNA identification

An optimized stepwise filtering workflow that based on our previous studies was used to identify lncRNAs (Jiang et al., 2016; Jiang and Kong, 2020). Briefly, raw data was processed by FastQC (Andrews, 2010) to remove low-quality reads. Stringtie (Pertea et al., 2015) was used for transcript assembly. Transcripts with class code “i” “j” “o” “u” “x”, exon number ≥ 2, and length over 200bp were retained and blast against annotated lncRNAs of rat genome (ALRG) to eliminate redundances. The transcripts were blast to pfam database (Bateman et al., 2004) and assessed by coding potential evaluation tools include CPC (Kong et al., 2007), CNCI (Sun et al., 2013) and CPAT (Wang et al., 2013), respectively. Transcripts that commonly evaluated as “noncoding” by the three analyses were defined as novel lncRNAs. ALRG profiles were acquired from http://ftp.ensembl.org/pub/release-87/fasta/rattus_norvegicus/dna/Rattus_norvegicus.Rnor_6.0.dna.toplevel.fa.gz.

### Novel circRNA identification

Candidate circRNA were identified using two tools include find_circ (Memczak et al., 2013) and CIRCexplorer (Zhang et al., 2014) following the standard tutorials with default parameters. Only transcripts that recognized as circRNA by both find_circ and CIRCexplorer were subjected for further analysis. Spliced reads per billion mapping (SRPBM) value for circRNA was calculated through: SRPBM = $\frac{\text{Circular}\;\text{reads}\;*\;10^{9}}{\text{Total}\;\text{mapped}\;\text{reads}\;*\;\text{read}\;\text{length}}$

### Prediction of interactions between circRNAs and miRNAs

The potential interactions between circRNAs and miRNAs were predicted by miRanda (John et al., 2004) using default parameters.

### Identification of differentially expressed PCGs and lncRNAs

RPKM (Reads per Kilobase per Million Reads) was calculated *via* formula: RPKM = $\frac{\text{Total exon reads }}{\text{mapped reads}\left(\text{millions}\right)\;*\;\text{exon}\;\text{length}\left(\text{kb}\right)}$ . By comparing the RPKM values, thresholds of |log(Fold change)| > 1 and p-value< 0.05 were set to define significantly differentially expressed genes. False discovery rate (FDR) was used for adjusting p-value. Unsupervised clustering was employed to uncover unknown relationships among genes and biological samples.

### Weighted gene co-expression network analysis analysis

WGCNA analysis was performed following its official tutorial (Langfelder and Horvath, 2008). Briefly, the normalized FPKM values of PCGs and lncRNAs were pooled and generated to adjacency matrix and subjected to “dynamicTreeCut” package (Langfelder et al., 2008) to filter out outliner samples. Then we used “pickSoftThreshold” function to calculate soft power values for predicting block-wise modules.

### Gene enrichment analysis

Gene ontology (GO) and Kyoto Encyclopedia of Genes and Genomes (KEGG) pathway mapping are useful strategies for gene functional classification (Kanehisa, 2019; Gene Ontology Consortium, 2021). Genes were classified to Gene Ontology and KEGG terms *via* online tool DAVID (https://david.ncifcrf.gov/) with default parameters.

## Results

### Renal pathological changes

**Figure 1:**
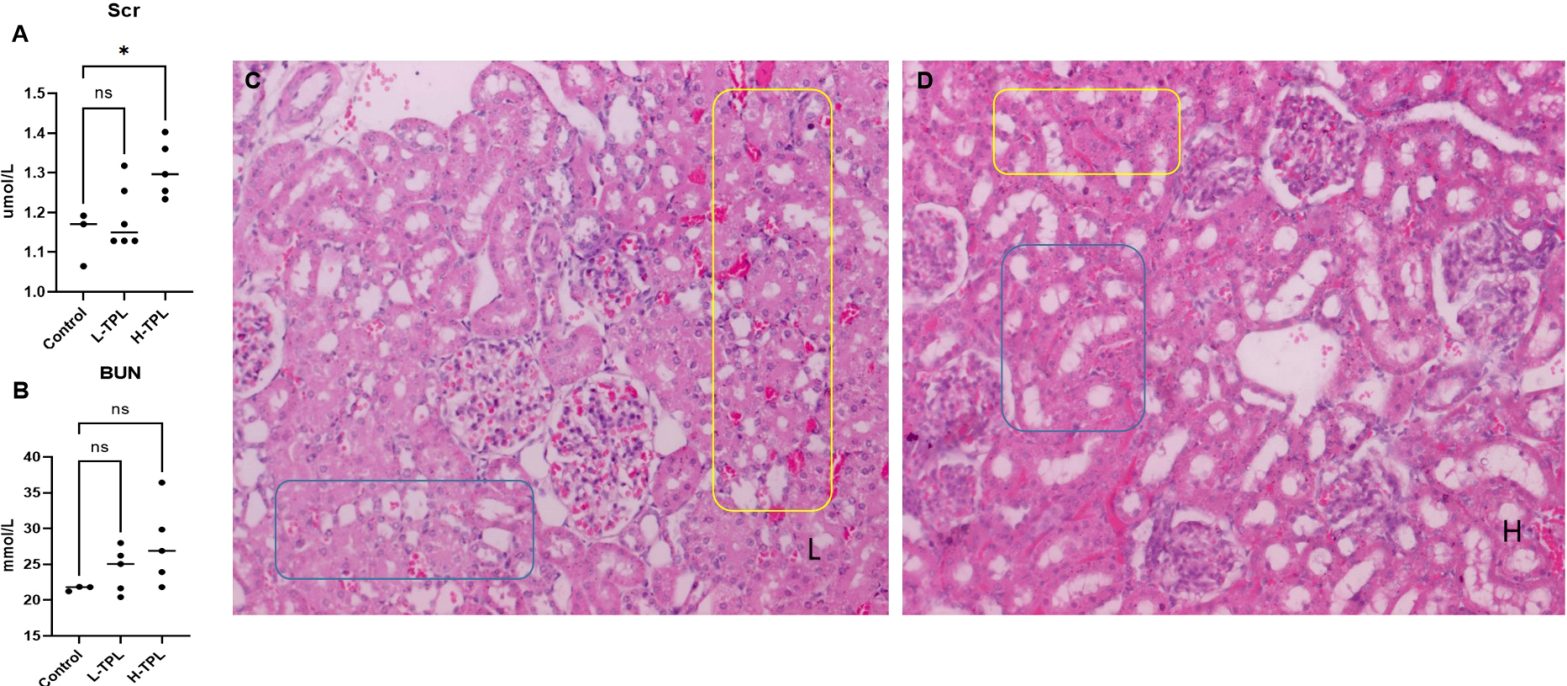
Serum creatinine (Scr), BUN and H & E staining in TPL treated rats. **(A)** Scr levels were significantly elevated in H-TPL groups; **(B)** BUN levels showed slightly but not statistically significant changes in L-TPL and H-TPL rats comparing with Ctrl group; **(C, D)** Representative captures of H & E staining of L-TPL **(C)** and H-TPL **(D)** kidneys, the yellow box is renal congestion and blue box is renal tubules atrophy. * indicates p < 0.05; ns indicates not significant.

The serum creatinine (Scr) and blood urea nitrogen (BUN) were tested and we found that H-TPL rats showed significant higher Scr (p-value = 0.0189) than Ctrl and L-TPL, indicated that treatment of high dosage of TPL induced declined renal function (**Figures 1A, B**). H & E staining found that both L-TPL and H-TPL rats exhibited tubular atrophy and renal blood vessel congestion comparing with Ctrl group **(** **Figures 1C, D** **),**. A total of 10 representative views (3-4 views/rat, n=3) were selected for each group for renal tubular injury scores evaluations, non-significant differences was observed between L-TPL and H-TPL(**Figure S1, S2**). However, wider intercellular gap between renal tubules was observed in H-TPL than L-TPL (**Figures 1C, D**).

### Potential targets of TPL nephrotoxicity predicted by network pharmacology-based analysis

**Figure 2:**
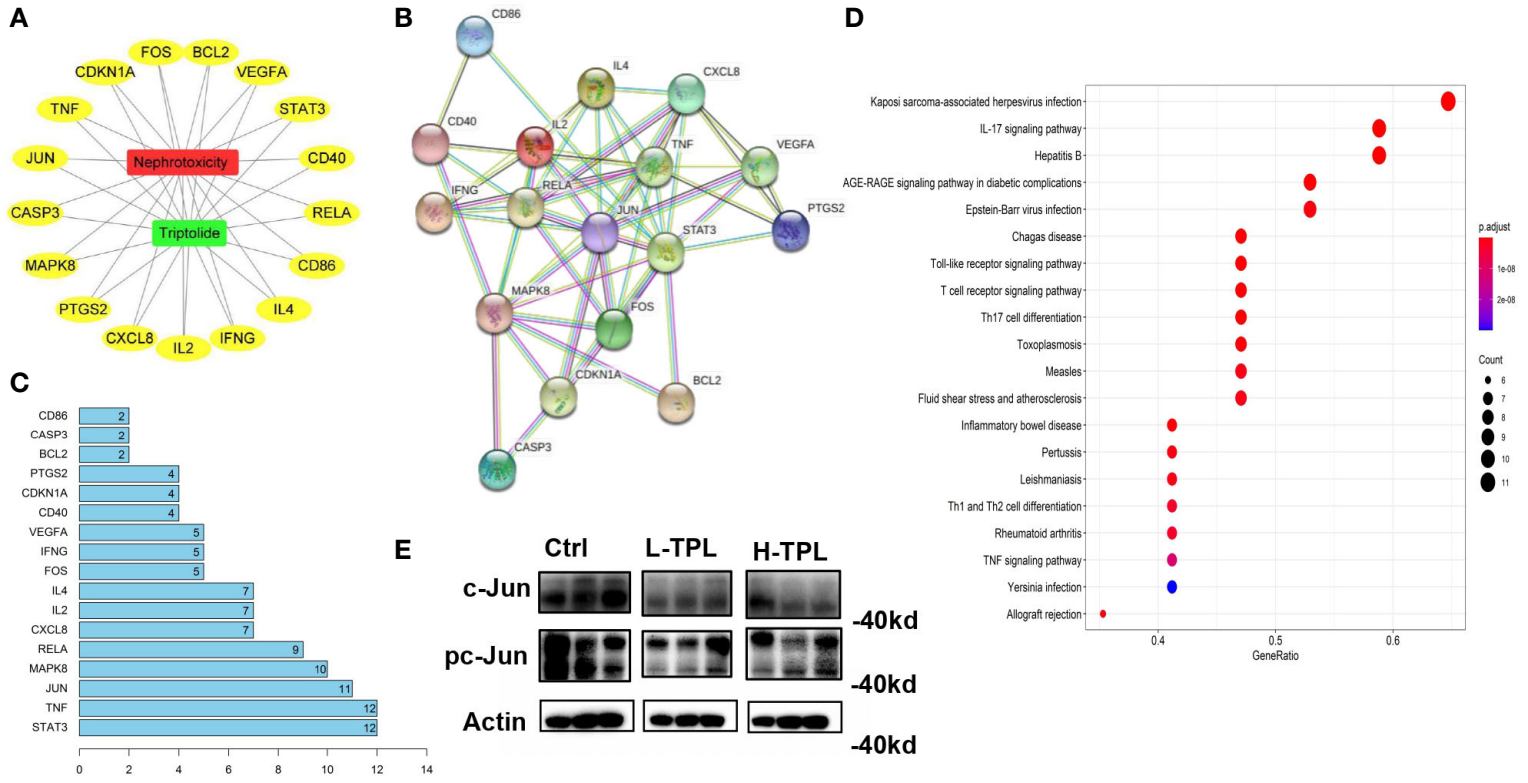
Target genes predicted to be associated with triptolide nephrotoxicity by network pharmacology-based analysis. **(A)** Visulization of interpalys between TPL and its predicted targets; **(B)** PPI network of TPL renal toxicity candidate target genes; **(C)** List of top 15 target genes ranked by contribution scores and they were regarded as candidate targeted genes related to TPL renal toxicity (CTGs-TPL); **(D)** KEGG terms of potential targets by gene enrichment analysis; **(E)** Western-blot assay of c-Jun and pc-Jun in Ctrl, L-TPL and H-TPL groups (from left to right, n = 3/group).

Network pharmacology-based analysis was performed to predict potential targets related with TPL renal toxicity. A total of 537 targets were predicted by GeneCards, OMIM, and DRUGBANK databases and 31 targets by the TCMSP database, we found that 17 were overlapped. The visualization of the interplay among TPL, nephrotoxicity and the 17 overlapped targets (OTs) was shown in **Figures 2A, B** by cytoscape3 (Shannon et al., 2003). These OTs were ported to STRING database to acquire their protein-protein interaction (PPI) pairs (**Figure 2B**). Contribution score (CS) was used to assess the importance of interested genes in contributing TPL renal toxicity, it is the number of gene nodes that correlated with each overlapped target (OT) within the PPI network. The contribution scores of 17 OTs were shown in **Figure 2C**, among which STAT3, TNF and JUN rank the top 3 genes with ≥ 11 gene nodes. By ranking the CSs, genes with CSs ≥ 4 were regarded as candidate targeted genes related to TPL renal toxicity (CTGs-TPL) (**Figure 2C**). Potential targets were subjected gene enrichment analysis and the top KEGG terms were shown in **Figure 2D**. Western-blot (WB) assay was employed to validate the association between the top-ranked CTGs-TPL include Stat3 and c-Jun, it showed that c-Jun were decreased in both L-TPL and H-TPL rats (**Figure 2E**), suggesting that c-Jun is a potential target of TPL. In addition, the activation status of c-Jun, the phosphorylated c-Jun (pc-Jun) was also inhibited slightly (**Figure 2E**), Considered the vital importance of Stat3 in renal function, two key factors that play essential roles in the upstream and/or downstream of Stat3 signaling include Jak2 and IL17 were selected to investigate their expression levels by WB assays although Stat3 and phosphorylated-Stat3 (pStat3) showed non-significant changes with TPL treatment (**Figure S3**). Nevertheless, neither Jak2/phosphorylated-Jak2 (p-Jak2) nor IL17 showed response to TPL treatment (**Figure S2**), demonstrating that TPL may not serve as a potential ligand for either Stat3/IL17 or Stat3/Jak2 signaling in the process of TPL nephrotoxicity.

**Figure 3:**
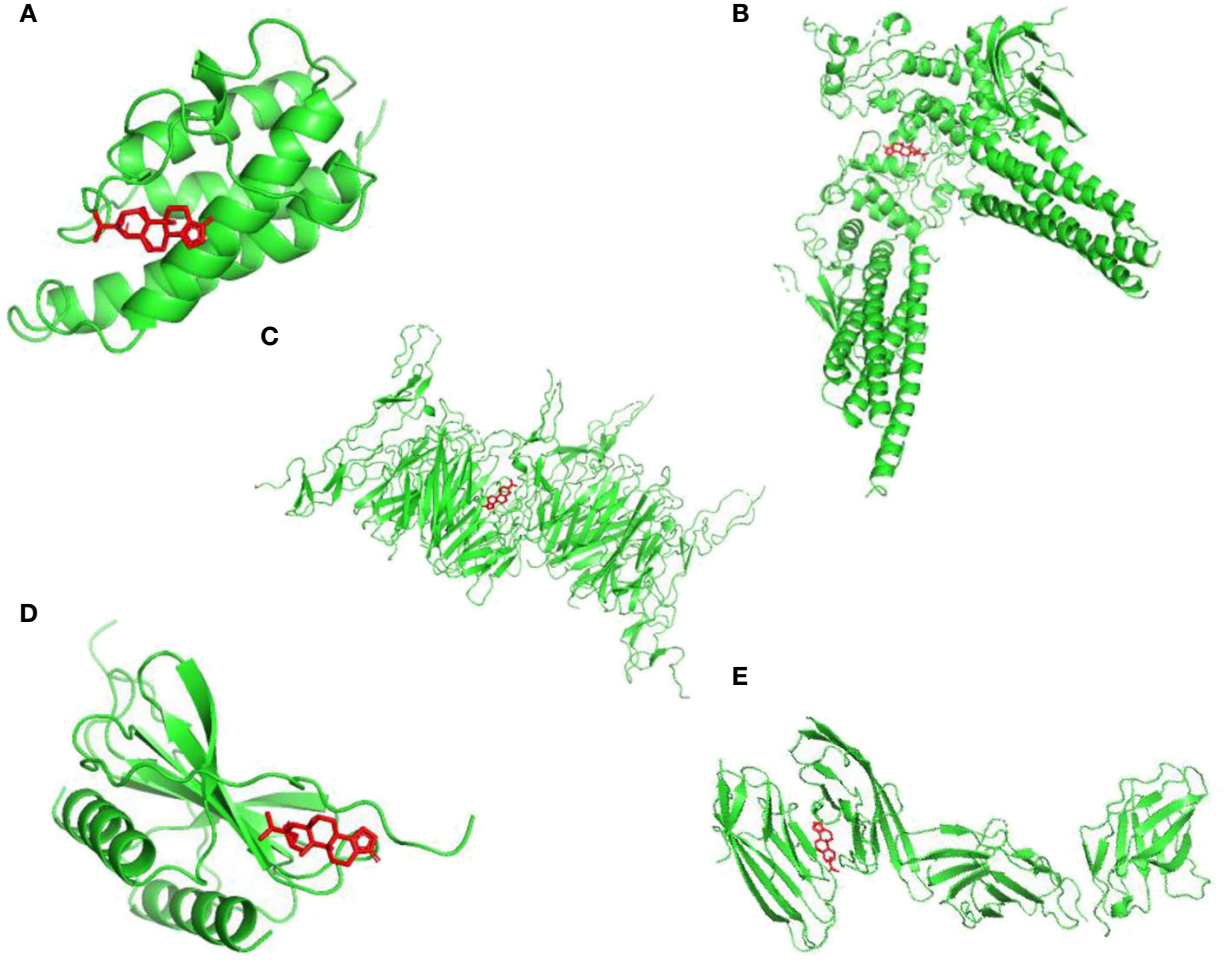
Visualizations of the interactions between targets and TPL by molecular docking analysis. **(A)** IL4; **(B)** STAT3; **(C)** CD40; **(D)** CXCL8; **(E)** CD86.

To discovery the structure-based associations between identified targets and TPL,

a molecular docking based strategy was applied to predict the ligand-target interactions between TPL and interested targets include CD86, IL4, CXCL8, STAT3 and CD40. Their interactions were visualized in **Figure 3**.

### Dysregulated protein coding genes

**Figure 4:**
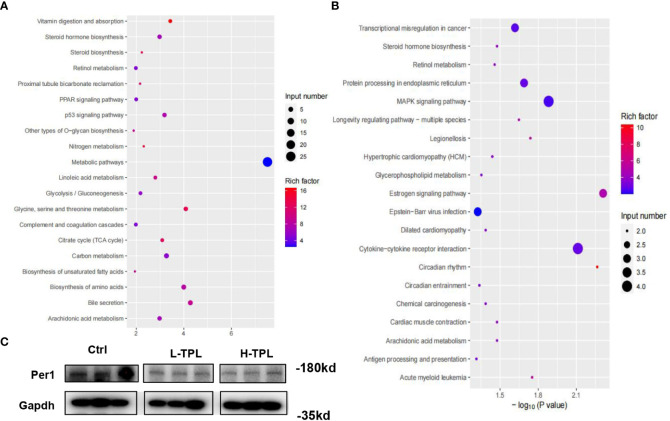
Identification of significant dysregulated genes in the renal tissues of H-TPL rats. **(A, B)** KEGG enrichment analysis of significant overexpressed **(A)** and downexpressed genes **(B, C)** Western-blot assay of Per1 in Ctrl, L-TPL and H-TPL groups.

As H-TPL rats showed aggravated renal pathological changes with wider intercellular gap between renal tubules, we selected H-TPL renal tissues instead of L-TPL for deep RNA-seq to elucidate the underlying molecular mechanisms of TPL induced renal toxicity. A total of 178 up-regulated and 152 downexpressed PCGs were obtained (**Table S2**). The gene enrichment analysis (GEA) indicates that up-regulated PCGs are classified (rich factor > 10) to vitamin digestion and absorption (hsa04977), Nitrogen metabolism (hsa00910), glycine, serine and threonine metabolism (hsa00260), steroid biosynthesis (hsa00100), citrate cycle (TCA cycle) (hsa00020) and proximal tubule bicarbonate reclamation (hsa04964) (**Figure 4A**). The downexpressed PCGs were significantly enriched in circadian rhythm (hsa04710) signaling (rich factor > 10) (**Figure 4B**). Co-expression correlation of protein-protein pairs was calculated by WGCNA. The networks of protein-protein interaction (PPI) were shown in **Figure S4**, the core genes include Ptcd3 and Cdk1.

Next to liver, kidney exerts the second most robust rhythms of circadian gene expression (Zhang et al., 2014; Myung et al., 2019). Among these dysregulated PCGs in H-TPL rats, we noticed that multiple circadian genes such as Per1, Per2, Per3 and Cry were significantly downexpressed. Previous study found that Per1 in kidney is important for renal sodium handling and necessary for maintaining homeostasis (Douma et al., 2022); therefore, Per1 was selected and validated by western blot assay, it showed that Per1 was decreased in TPL treated kidney (**Figure 4C**). Although the roles of circadian genes are unknown in the mechanisims of TPL renal toxicity, our results suggested that TPL may relate with the circadian pace of kidney function.

It is interesting that we noticed that *c-Jun* was not among the significant dysregulated genes by TPL treatment, suggesting that c-Jun may involve in TPL toxicity in renal tissues *via* post-transcriptional regulation.

### Significantly differentially expressed lncRNAs

**Figure 5:**
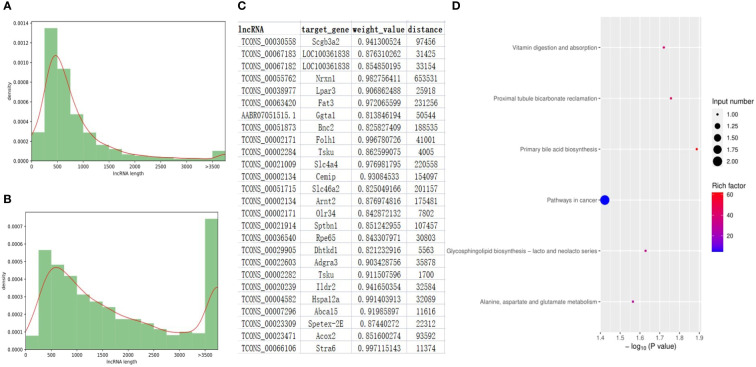
Features of lncRNA genes in the renal tissues of H-TPL rats. **(A, B)** Comparison of lengths between ALRGs **(A)** and novel lncRNAs **(B, C)** Pairs of SDElncs and 27 candidate target genes predicted by location and WGCNA; **(D)** KEGG terms of the 27 target genes.

After a strict filtering pipeline, a total of 6061 novel lncRNAs were identified and combined with the annotated lncRNAs of rat genome (ALRG) for next-step analysis. The length distribution and exon number density plots were shown in **Figure 5A, B** and **Figure S5**. The majority of novel lncRNAs and ALRG own 2 exons. Unlike ALRG that generally enriched in 200 - 500bp, the length of novel lncRNAs are mostly distributed in 200 - 500, 500 - 1000 and > 3500 bp (**Figures 5A, B**). A total of 131 up- and 119 down-expressed lncRNAs were identified as significantly differentially expressed lncRNAs (SDElncs) in H-TPL (p-value< 0.05). Increasing studies have demonstrated that lncRNAs usually owns the capacity to regulate their nearby genes (Statello et al., 2021). To illustrate the potential roles of SDElncs, genes that locate within 100kb of SDElncs were acquired and own strong correlations with SDElncRNAs (weight value > 0.8) were defined as target genes (**Figure 5C**). A total of 26 genes were identified and subjected for GEA. We surprisingly found target genes, alike with the dysregulated PCGs, were also enriched in vitamin digestion and absorption (hsa04977) and metabolic related pathways such as Alanine, aspartate and glutamate metabolism (hsa00250) (**Figure 5D**), which suggesting that abnormal metabolism of amino acids was potentially related with TPL nephrotoxicity.

### CircRNA signatures

A total of 1529 high-quality novel circRNAs were identified. By calculating the SRPBM values, only 7 circRNAs were found deferentially expressed in H-TPL rats with p-value< 0.05 (**Table S1** **)**. As circRNAs can be served as miRNA sponges, we utilized miRanda tool (Enright et al., 2003) to predict the potential connections between dysregulated circRNAs and miRNAs. Ranking by tot scores, the top 10 circRNA-miRNA pairs were shown in **Table 1**. Previous studies reveled that miR-207 was up-regulated in renal and urine of rats with renal fibrosis and decreased in ischemia-reperfusion injury (IRI) model of mouse (Wei et al., 2010; Shi et al., 2018). We found that both circ: Chr 6:124934385-124981303 and circ: Chr11: 66774701-66795896 are strongly targeted with miR-207 (Tot scores > 1000), suggesting these two circRNAs may be involved with mi-207 related renal function regulation although the deep mechanisms is unknown.

**Table 1:** The top 10 miRNA-circRNA interaction pairs ranked by Tot_scores.

miRNA	circRNA	Tot_Scores	Positions
miR-320-5p	6:124934385-124981303	1973	1398 1911 2510 1211 1323 2813 1581 2198 1472 2378 2300 1608 1124
miR-3557-5p	11:66774701-66795896	1334	3240 7004 6394 2685 2835 1684 5020 5102 5855
miR-207	6:124934385-124981303	1253	1577 527 1217 1817 210 1398 1127 722
miR-337-3p	11:66774701-66795896	1203	1929 7259 580 7055 6716 1893 3024 587
miR-207	11:66774701-66795896	1074	4055 4779 6296 1763 5711 218 6812
miR-127-5p	11:66774701-66795896	1050	125 5264 4301 1792 5339 6845 2597
miR-3575	5:151944768-151947717	1048	287 312 634 414 579 464 537
miR-3584-5p	4:51682050-51690235	1011	456 539 426 398 366 561 651
miR-3551-5p	9:37960098-38013690	948	278 2478 1751 541 5371 822
miR-103-1-5p	1:134783877-134848903	925	1 3942 1332 3830 2641 3256

## Discussion

TPL has been applied as an useful compound for treatment of multiple renal diseases for decades; however its toxicity largely limited its clinical practice. Metabolites has been studied in a broad field such as screening for new therapeutic targets, discovery and validation of disease biomarkers. Multitude studies have applied metabonomics technology to investigate TPL, the regulating mechanisms and the toxicities (Du et al., 2014; Li et al., 2019); however, the transcriptional changes of TPL nephrotoxicity were rarely reported. In this study, a combined approach of network pharmacology method and RNA-seq was used to elucidate the molecular mechanisms of TPL nephrotoxicity. RNA-seq analysis found that a series of circadian genes, such as Per1-3, were significantly dysregulated in renal tissues along with H-TPL treatment. Per1-3 are closely related with renal rhythm. Per1 acts as a circadian clock transcription factor and was regulated by aldosterone, a steroid hormone increases blood pressure *via* elevating blood volume and Na+ retention (Douma et al., 2022). Myung et al. demonstrated that the mouse kidney of adenine diet induced chronic kidney disease (CDK) model displayed disorganization of Per2 expression (Myung et al., 2019). Per3 exerts dynamic expression patterns in pan renal carcinoma (Liu et al., 2021). Through western blot assay, we validated that Per1 was decreased along with TPL treatment, suggesting that Per1 is involved in the regulation of TPL nephrotoxicity.

For identifying candidate targets of TPL nephrotoxicity by network pharmacology based analysis, Huang et al. employed GeneMANIA database and screened out 39 direct-targets in male rats (Huang et al., 2019). Although there is no evidence suggested that TPL toxicity has sexual difference, our study utilized female rats to identify TPL regulated proteins and found that female rats seem to tolerant the renal toxicty under both L-TPL and H-TPL treatment with low renal injure rate. Comparing with Huang’s study, a more strict filtering standard and different databases were used and we gained highly consistent targets. Our further investigations by western-blots validated that c-Jun protein is a potential target of TPL. c-Jun protein is a widely expressed transcription factor associated with a variety of diseases include human renal diseases (Blau et al., 2012). In glomerular and tubular cells, c-Jun was activated and its activation involves in the regulation of renal inflammation and/or fibrosis (De Borst et al., 2007).

RNA-seq and network pharmacology are different techniques to elucidate relevant candidate molecular targets from two perspectives. Network pharmacology is a novel approach that widely applied for discovering the targets involved in the process of TCM compounds or modern drugs treatment in a specific disease *via* integrating biomedical, pharmacological and computational approaches while RNA-seq can gain us a cohort of genes with differential expression directly. A combination of these two techniques definitely provide a more comprehensive knowledge of molecular mechanisms in the process of TPL induced renal toxicity. In this study, Pe1 and c-Jun are two candidates related with TPL nephrotoxicity identified by these two analyses, respectively. Although little evidence has been implied on the connections between Per1 and c-Jun and the mechanisms among c-Jun, Per1, TPL and nephrotoxicity are remain explored, our study suggested that c-Jun protein and Per1 are possibly to be involved in TPL induced renal toxicity *via* two independent pathways.

## Data availability statement

The datasets presented in this study can be found in online repositories. The names of the repository/repositories and accession number(s) can be found in the article/**Supplementary Material**.

## Ethics statement

The animal study was reviewed and approved by Shenzhen PKU-HKUST Medical Center.

## Author contributions

S-ML, S-DY, J-JJ, and Y-ML conceived and designed this study. Y-ML, M-YW, S-TL, BW, J-HL, Y-YL, HC, L-LZ and J-JJ performed all the experiments and data analysis. J-JJ, S-DY and S-ML wrote and revised this manuscript. All authors contributed to the article and approved the submitted version.

## References

[B1] Anastasiadou E. Jacob L. S. Slack F. J. (2018). Non-coding RNA networks in cancer. Nat. Rev. Cancer 18 (1), 5–18. doi: 10.1038/nrc.2017.99 29170536 PMC6337726

[B2] Andrews S. (2010). FastQC: a quality control tool for high throughput sequence data. Available at: http://www.bioinformatics.babraham.ac.uk/projects/fastqc.

[B3] Bateman A. Coin L. Durbin R. Finn R. D. Hollich V. Griffiths-Jones S.. (2004). The pfam protein families database. Nucleic Acids Res. 32 (suppl_1), D138–D141. doi: 10.1093/nar/gkh121 14681378 PMC308855

[B4] Bhatti G. K. Khullar N. Sidhu I. S. Navik U. S. Reddy A. P. Reddy P. H.. (2021). Emerging role of non-coding RNA in health and disease. Metab. Brain Dis. 36 (6), 1119–1134. doi: 10.1007/s11011-021-00739-y 33881724 PMC8058498

[B5] Blau L. Knirsh R. Ben-Dror I. Oren S. Kuphal S. Hau P.. (2012). Aberrant expression of c-jun in glioblastoma by internal ribosome entry site (IRES)-mediated translational activation. Proc. Natl. Acad. Sci. 109 (42), E2875–E2884. doi: 10.1073/pnas.1203659109 23027969 PMC3479486

[B6] Chen T. Chen X. Zhang S. Zhu J. Tang B. Wang A.. (2021). The genome sequence archive family: Toward explosive data growth and diverse data types. Genomics Proteomics Bioinf. 19 (4), 578–583. doi: 10.1016/j.gpb.2021.08.001 PMC9039563 34400360

[B7] Cheng Y. Zhao Y. Zheng Y. (2021). Therapeutic potential of triptolide in autoimmune diseases and strategies to reduce its toxicity. Chin. Med. 16 (1), 1–21. doi: 10.1186/s13020-021-00525-z 34743749 PMC8572577

[B8] De Borst M. Prakash J. Melenhorst W. Van Den Heuvel M. Kok R. Navis G.. (2007). Glomerular and tubular induction of the transcription factor c-jun in human renal disease. J. Pathol. 213 (2), 219–228. doi: 10.1002/path.2228 17891746

[B9] Di Agostino S. Riccioli A. De Cesaris P. Fontemaggi G. Blandino G. Filippini A.. (2020). Circular RNAs in embryogenesis and cell differentiation with a focus on cancer development. Front. Cell Dev. Biol. 8, 389. doi: 10.3389/fcell.2020.00389 32528957 PMC7266935

[B10] Douma L. G. Costello H. M. Crislip G. R. Cheng K.-Y. Lynch I. J. Juffre A.. (2022). Kidney-specific KO of the circadian clock protein PER1 alters renal na+ handling, aldosterone levels, and kidney/adrenal gene expression. Am. J. Physiology-Renal Physiol. 322 (4), F449–F459. doi: 10.1152/ajprenal.00385.2021 PMC9169971 35129370

[B11] Du F. Liu Z. Li X. Xing J. (2014). Metabolic pathways leading to detoxification of triptolide, a major active component of the herbal medicine tripterygium wilfordii. J. Appl. Toxicol. 34 (8), 878–884. doi: 10.1002/jat.2906 23836259

[B12] Enright A. John B. Gaul U. Tuschl T. Sander C. Marks D. (2003). MicroRNA targets in drosophila. Genome Biol. 4 (11), 1–27. doi: 10.1186/gb-2003-5-1-r1 PMC395733 14709173

[B13] Fan D. Guo Q. Shen J. Zheng K. Lu C. Zhang G.. (2018). The effect of triptolide in rheumatoid arthritis: from basic research towards clinical translation. Int. J. Mol. Sci. 19 (2), 376. doi: 10.3390/ijms19020376 29373547 PMC5855598

[B14] Fatica A. Bozzoni I. (2014). Long non-coding RNAs: new players in cell differentiation and development. Nat. Rev. Genet. 15 (1), 7–21. doi: 10.1038/nrg3606 24296535

[B15] Ge M.-X. Jiang J.-J. Yang L.-Q. Yang X.-L. He Y.-H. Li G.-H.. (2022). Specific gain and loss of Co-expression modules in long-lived individuals indicate a role of circRNAs in human longevity. Genes 13 (5), 749. doi: 10.3390/genes13050749 35627134 PMC9140997

[B16] Gene Ontology Consortium. (2021). The gene ontology resource: enriching a GOld mine. Nucleic Acids Res. 49 (D1), D325–d334. 33290552 10.1093/nar/gkaa1113 PMC7779012

[B17] He A. T. Liu J. Li F. Yang B. B. (2021). Targeting circular RNAs as a therapeutic approach: Current strategies and challenges. Signal transduction targeted Ther. 6 (1), 1–14. doi: 10.1038/s41392-021-00569-5 PMC8137869 34016945

[B18] Huang W. Liu C. Xie L. Wang Y. Xu Y. Li Y. (2019). Integrated network pharmacology and targeted metabolomics to reveal the mechanism of nephrotoxicity of triptolide. Toxicol. Res. 8 (6), 850–861. doi: 10.1039/c9tx00067d PMC7017871 32110379

[B19] Jiang J.-J. Cheng L.-H. Wu H. He Y.-H. Kong Q.-P. (2016). Insights into long noncoding RNAs of naked mole rat (Heterocephalus glaber) and their potential association with cancer resistance. Epigenet. chromatin 9 (1), 1–10. doi: 10.1186/s13072-016-0101-5 PMC5103457 27833660

[B20] Jiang J. Cheng L. Yan L. Ge M. Yang L. Ying H.. (2021). Decoding the role of long noncoding RNAs in the healthy aging of centenarians. Briefings Bioinf. 22 (5), bbaa439. doi: 10.1093/bib/bbaa439 33517370

[B21] Jiang J.-J. Kong Q.-P. (2020). Comparative analysis of long noncoding RNAs in long-lived mammals provides insights into natural cancer-resistance. RNA Biol. 17 (11), 1657–1665. doi: 10.1080/15476286.2020.1792116 32635806 PMC7567513

[B22] John B. Enright A. J. Aravin A. Tuschl T. Sander C. Marks D. S.. (2004). Human microRNA targets. PloS Biol. 2 (11), e363. doi: 10.1371/journal.pbio.0020363 15502875 PMC521178

[B23] Kanehisa M. (2019). Toward understanding the origin and evolution of cellular organisms. Protein Sci. 28 (11), 1947–1951. doi: 10.1002/pro.3715 31441146 PMC6798127

[B24] Kong L. Zhang Y. Ye Z.-Q. Liu X.-Q. Zhao S.-Q. Wei L.. (2007). CPC: assess the protein-coding potential of transcripts using sequence features and support vector machine. Nucleic Acids Res. 35 (suppl_2), W345–W349. doi: 10.1093/nar/gkm391 17631615 PMC1933232

[B25] Langfelder P. Horvath S. (2008). WGCNA: an r package for weighted correlation network analysis. BMC Bioinf. 9 (1), 1–13. doi: 10.1186/1471-2105-9-559 PMC2631488 19114008

[B26] Langfelder P. Zhang B. Horvath S. (2008). Defining clusters from a hierarchical cluster tree: the dynamic tree cut package for r. Bioinformatics 24 (5), 719–720. doi: 10.1093/bioinformatics/btm563 18024473

[B27] Li J. Huang Y. Zhao S. Guo Q. Zhou J. Han W.. (2019). Based on network pharmacology to explore the molecular mechanisms of astragalus membranaceus for treating T2 diabetes mellitus. Ann. Trans. Med. 7 (22), 633. doi: 10.21037/atm.2019.10.118 PMC6944577 31930034

[B28] Li R. Li Y. Liang X. Yang L. Su M. Lai K. P. (2021). Network pharmacology and bioinformatics analyses identify intersection genes of niacin and COVID-19 as potential therapeutic targets. Briefings Bioinf. 22 (2), 1279–1290. doi: 10.1093/bib/bbaa300 PMC7717147 33169132

[B29] Li C. Li Z. Zhang T. Wei P. Li N. Zhang W.. (2019). 1H NMR-based metabolomics reveals the antitumor mechanisms of triptolide in BALB/c mice bearing CT26 tumors. Front. Pharmacol. 10, 1175. doi: 10.3389/fphar.2019.01175 31680959 PMC6798008

[B30] Li S. Zhang B. (2013). Traditional Chinese medicine network pharmacology: theory, methodology and application. Chin. J. Natural Medicines 11 (2), 110–120. doi: 10.1016/S1875-5364(13)60037-0 23787177

[B31] Li S. Zhang Z. Q. Wu L. J. Zhang X. G. Li Y. D. Wang Y. Y. (2007). Understanding ZHENG in traditional Chinese medicine in the context of neuro-endocrine-immune network. IET Syst. Biol. 1 (1), 51–60. doi: 10.1049/iet-syb:20060032 17370429

[B32] Li S. Zhang B. Zhang N. (2011). Network target for screening synergistic drug combinations with application to traditional Chinese medicine. BMC Syst. Biol. 5 (1), S10. doi: 10.1186/1752-0509-5-S1-S10 PMC3121110 21689469

[B33] Liu S. Cheng Y. Wang S. Liu H. (2021). Circadian clock genes modulate immune, cell cycle and apoptosis in the diagnosis and prognosis of pan-renal cell carcinoma. Front. Mol. Biosci. 8. doi: 10.3389/fmolb.2021.747629 PMC8717949 34977153

[B34] Matsui M. Corey D. R. (2017). Non-coding RNAs as drug targets. Nat. Rev. Drug Discovery 16 (3), 167–179. doi: 10.1038/nrd.2016.117 27444227 PMC5831170

[B35] Memczak S. Jens M. Elefsinioti A. Torti F. Krueger J. Rybak A.. (2013). Circular RNAs are a large class of animal RNAs with regulatory potency. Nature 495 (7441), 333–338. doi: 10.1038/nature11928 23446348

[B36] Meng C. Zhu H. Song H. Wang Z. Huang G. Li D.. (2014). Targets and molecular mechanisms of triptolide in cancer therapy. Chin. J. Cancer Res. 26 (5), 622. doi: 10.3978/j.issn.1000-9604.2014.09.01 25400429 PMC4220249

[B37] Myung J. Wu M.-Y. Lee C.-Y. Rahim A. R. Truong V. H. Wu D.. (2019). The kidney clock contributes to timekeeping by the master circadian clock. Int. J. Mol. Sci. 20 (11), 2765. doi: 10.3390/ijms20112765 31195684 PMC6600447

[B38] Niu W.-H. Wu F. Cao W.-Y. Wu Z.-G. Chao Y.-C. Peng F. (2021). Liang c: Network pharmacology for the identification of phytochemicals in traditional Chinese medicine for COVID-19 that may regulate interleukin-6. Bioscience Rep. 41 (1), BSR20202583. doi: 10.1042/BSR20202583 PMC7809559 33146673

[B39] Noel P. Hussein S. Ng S. Antal C. E. Lin W. Rodela E.. (2020). Triptolide targets super-enhancer networks in pancreatic cancer cells and cancer-associated fibroblasts. Oncogenesis 9 (11), 1–12. doi: 10.1038/s41389-020-00285-9 33168807 PMC7653036

[B40] Noel P. Von Hoff D. D. Saluja A. K. Velagapudi M. Borazanci E. Han H. (2019). Triptolide and its derivatives as cancer therapies. Trends Pharmacol. Sci. 40 (5), 327–341. doi: 10.1016/j.tips.2019.03.002 30975442

[B41] Park S.-W. Kim Y. I. (2013). Triptolide induces apoptosis of PMA-treated THP-1 cells through activation of caspases, inhibition of NF-κB and activation of MAPKs. Int. J. Oncol. 43 (4), 1169–1175. doi: 10.3892/ijo.2013.2033 23900299

[B42] Pertea M. Pertea G. M. Antonescu C. M. Chang T.-C. Mendell J. T. Salzberg S. L. (2015). StringTie enables improved reconstruction of a transcriptome from RNA-seq reads. Nat. Biotechnol. 33 (3), 290–295. doi: 10.1038/nbt.3122 25690850 PMC4643835

[B43] Ru J. Li P. Wang J. Zhou W. Li B. Huang C.. (2014). et al: TCMSP: a database of systems pharmacology for drug discovery from herbal medicines. J. Cheminformatics 6 (1), 13. doi: 10.1186/1758-2946-6-13 PMC4001360 24735618

[B44] Shannon P. Markiel A. Ozier O. Baliga N. S. Wang J. T. Ramage D.. (2003). Cytoscape: a software environment for integrated models of biomolecular interaction networks. Genome Res. 13 (11), 2498–2504. doi: 10.1101/gr.1239303 14597658 PMC403769

[B45] Shi B.-H. Sun J.-Z. Zhang S. Shi J. (2018). Expression of miR-207 was up-regulated in renal and urine of rats with renal fibrosis. Basic Clin. Med. 38 (3), 308.

[B46] Statello L. Guo C.-J. Chen L.-L. Huarte M. (2021). Gene regulation by long non-coding RNAs and its biological functions. Nat. Rev. Mol. Cell Biol. 22 (2), 96–118. doi: 10.1038/s41580-020-00315-9 33353982 PMC7754182

[B47] Sun L. Luo H. Bu D. Zhao G. Yu K. Zhang C.. (2013). Utilizing sequence intrinsic composition to classify protein-coding and long non-coding transcripts. Nucleic Acids Res. 41 (17), e166–e166. doi: 10.1093/nar/gkt646 23892401 PMC3783192

[B48] Wang L. Park H. J. Dasari S. Wang S. Kocher J.-P. Li W. (2013). CPAT: Coding-potential assessment tool using an alignment-free logistic regression model. Nucleic Acids Res. 41 (6), e74–e74. doi: 10.1093/nar/gkt006 23335781 PMC3616698

[B49] Wei Q. Bhatt K. He H.-Z. Mi Q.-S. Haase V. H. Dong Z. (2010). Targeted deletion of dicer from proximal tubules protects against renal ischemia-reperfusion injury. J. Am. Soc. Nephrol. 21 (5), 756–761. doi: 10.1681/ASN.2009070718 20360310 PMC2865746

[B50] You L. Dong X. Ni B. Fu J. Yang C. Yin X.. (2018). Triptolide induces apoptosis through fas death and mitochondrial pathways in HepaRG cell line. Front. Pharmacol. 9, 813. doi: 10.3389/fphar.2018.00813 30093863 PMC6070613

[B51] Zhang R. Lahens N. F. Ballance H. I. Hughes M. E. Hogenesch J. B. (2014). A circadian gene expression atlas in mammals: implications for biology and medicine. Proc. Natl. Acad. Sci. 111 (45), 16219–16224. doi: 10.1073/pnas.1408886111 25349387 PMC4234565

[B52] Zhang L. Wang T. Li Q. Huang J. Xu H. Li J.. (2019). Fabrication of novel vesicles of triptolide for antirheumatoid activity with reduced toxicity *in vitro* and *in vivo* [Corrigendum]. Int. J. Nanomedicine 14, 2755–2756. doi: 10.2147/IJN.S104593 31114194 PMC6489557

[B53] Zhang X.-O. Wang H.-B. Zhang Y. Lu X. Chen L.-L. Yang L. (2014). Complementary sequence-mediated exon circularization. Cell 159 (1), 134–147. doi: 10.1016/j.cell.2014.09.001 25242744

[B54] Zhao S. Li S. (2012). A co-module approach for elucidating drug–disease associations and revealing their molecular basis. Bioinformatics 28 (7), 955–961. doi: 10.1093/bioinformatics/bts057 22285830

[B55] Zuo H. Zhang Q. Su S. Chen Q. Yang F. Hu Y. (2018). A network pharmacology-based approach to analyse potential targets of traditional herbal formulas: an example of yu ping feng decoction. Sci. Rep. 8 (1), 1–15. doi: 10.1038/s41598-018-29764-1 30061691 PMC6065326

