# Supplementary material for: Insights into the mechanisms of triptolide nephrotoxicity through network pharmacology-based analysis and RNA-seq
Source: Front Plant Sci. 2023 Mar 7;14:1144583. doi: 10.3389/fpls.2023.1144583 (PMC10027700; doi:10.3389/fpls.2023.1144583)
Supplement: Supplementary file 1 [file DataSheet_1.docx]

**Supplementary Files**

**Figure S1**

**
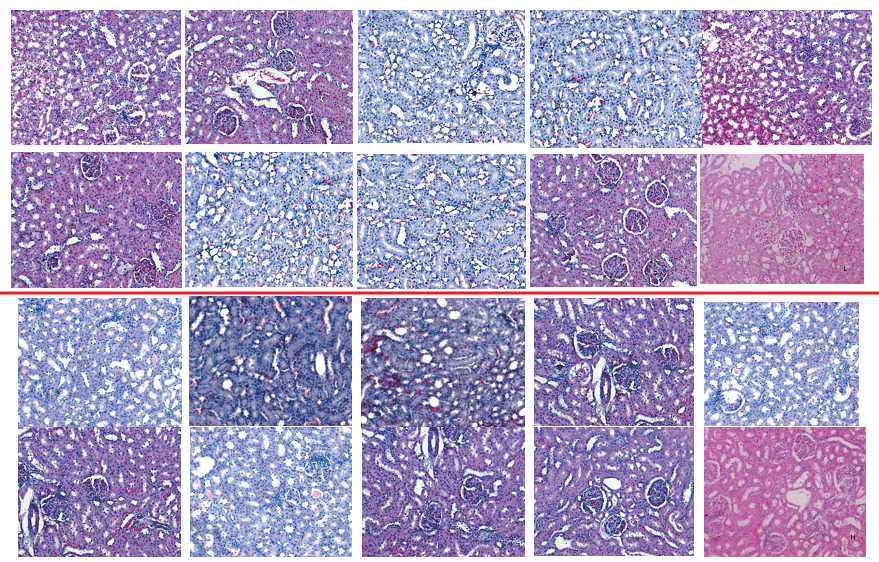
**

**Figure S1** Representative views used for generating renal tubular injury scores. The upper and lower panels are L-TPL and H-TPL groups, respectively.

**Figure S2**

**
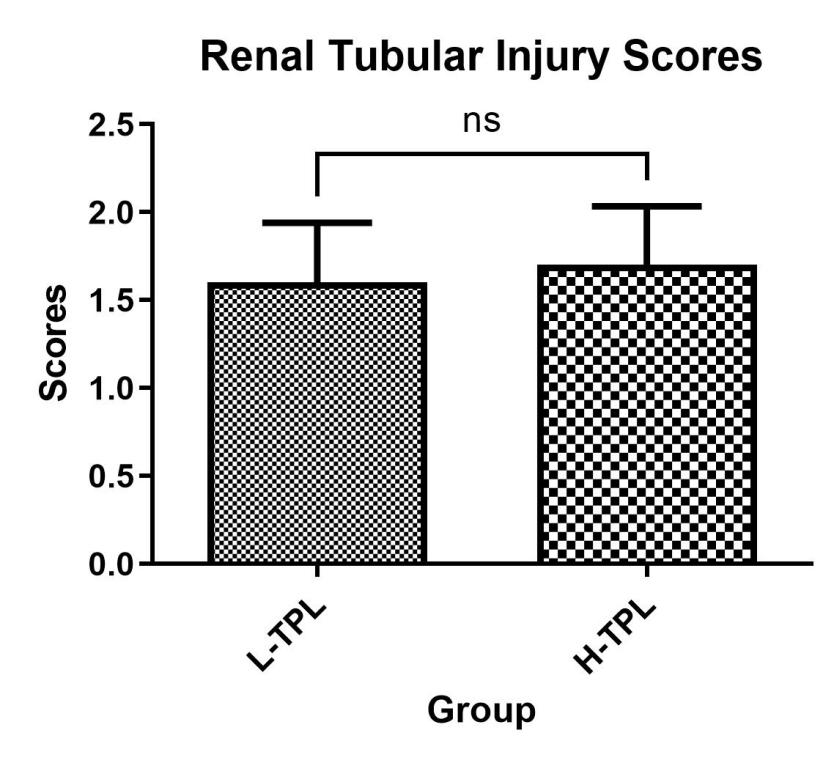
**

**Figure S2** Renal tubular injury scores of L-TPL and H-TPL groups of rats. The percentage of renal tubular injury area (%) was estimated to semi-quantitate the degree of renal tubular injury. The score was counted as following standards: 0 (none) ,1 point (<25%); 2 points (26%~50%); 3 points (51%~75%); 4 points (>75%) .

**Figure S3**


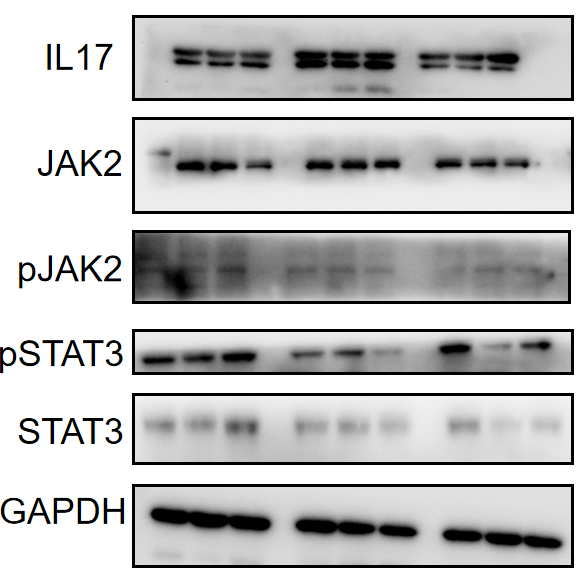


**Figure S3** Western-blot assays of IL17, Jak2, pJak2, Stat3 and pStat3 in Ctrl (left panel, n =3), L-TPL (middle panel, n = 3) and H-TPL (right panel, n = 3), Gapdh was employed as the reference. Non-significant change was observed among the three groups.

**FigureS4
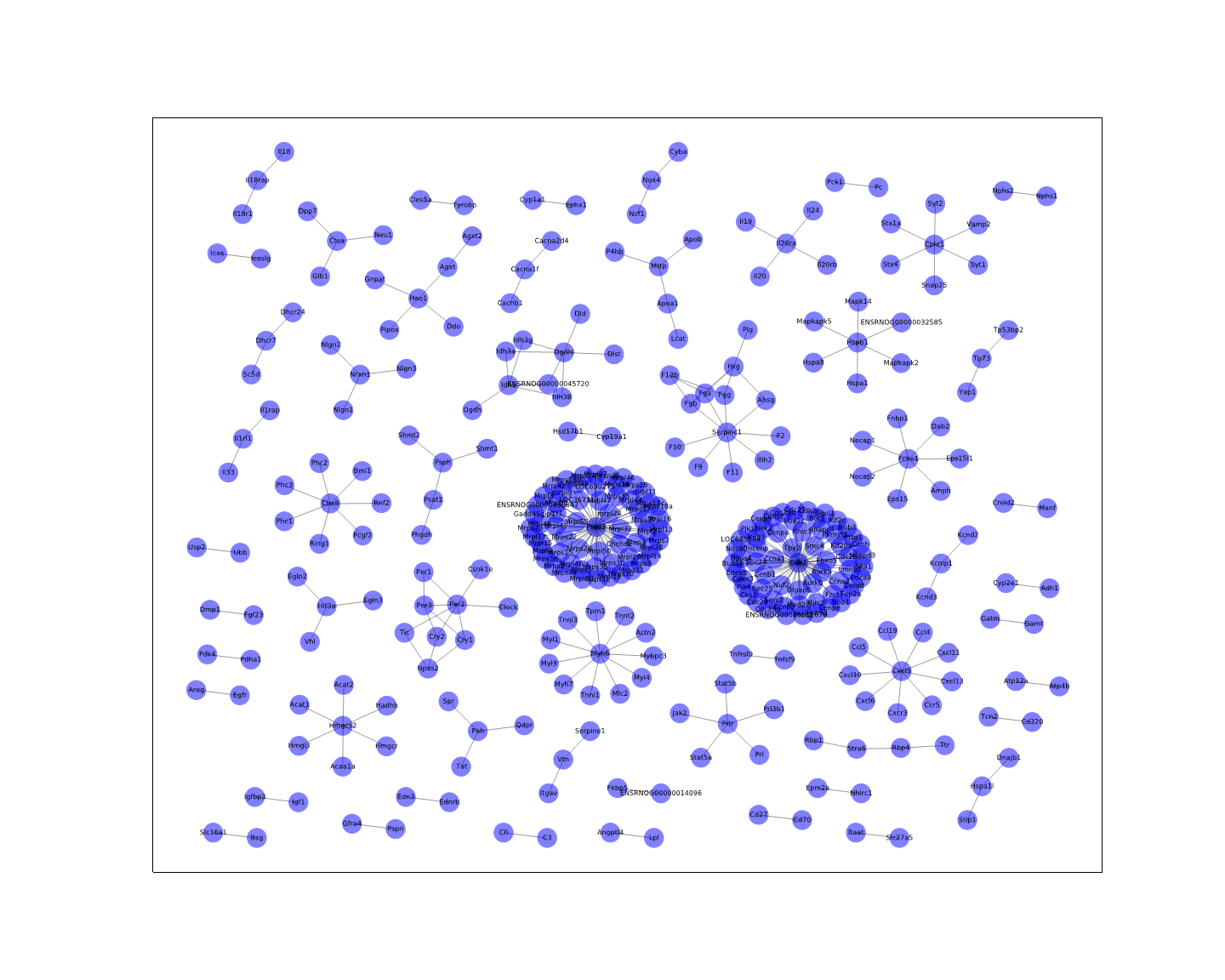
Figure S4.** Visualization of PPI networks of SDE-PCGs by cytoscape, hub genes with the most abundant nodes are Ptcd3 and Cdk1.

**FigureS5**


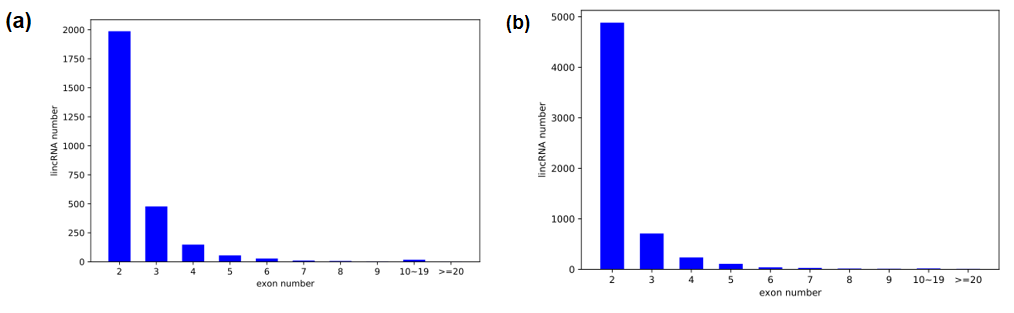


**Figure S5** Distribution of exon numbers of ALRGs (a) and novel lncRNA (b).

**Table S1.** List of antibody that we have used in this study

| Protein | Catlog No. |
| --- | --- |
| IL-17 | Sigma #PRS4887 |
| Stat3 | CST #9139S |
| p-Stat3 | CST #9145S |
| b-actin | CST #4970S |
| IL6 | abcam #ab9324 |
| c-Jun | abcam #ab40766 |
| pC-Jun | abcam #ab32385 |
| Gapdh | CST #2118S |
| Jak2 | abcam #ab108596 |
| pJak2 | CST #4406T |
| Per1 | Santa Cruz #sc398890 |

**Table S2.** FPKM values of dysregulated genes

| Geneid | Model_1 | Model_2 | Model_3 | Control_1 | Control_2 | Control_3 |
| --- | --- | --- | --- | --- | --- | --- |
| Epm2a | 2.515338435 | 1.650239367 | 1.980855706 | 2.668688011 | 4.790234829 | 6.979198273 |
| Arfgef3 | 15.55225905 | 7.204630152 | 24.41257179 | 6.885609798 | 6.117664429 | 7.43083022 |
| Il20ra | 1.31404997 | 0.091342547 | 1.67349112 | 0.098476526 | 0.118975194 | 0.206423176 |
| Vnn1 | 13.9621133 | 6.473159084 | 20.04956631 | 6.021342074 | 4.744081678 | 2.809393525 |
| Slc6a19 | 17.90596912 | 0.368043629 | 22.57081424 | 1.20950833 | 0.648788989 | 2.259128093 |
| Tcte3 | 1.613006683 | 1.781857648 | 2.291617482 | 4.985512568 | 4.925990472 | 3.246081046 |
| Hif3a | 1.23583271 | 2.046073054 | 0.649199141 | 2.068007048 | 4.385085707 | 2.25591042 |
| Cyp2b2 | 0.214500189 | 0.876730755 | 0.178034027 | 0.765165659 | 87.04504827 | 0.30326114 |
| Ccer2 | 1.292149138 | 0.548719592 | 0.758317058 | 1.725428103 | 1.786791089 | 3.026288861 |
| Arhgap33 | 0.215872171 | 0.342131249 | 0.234631002 | 0.517687259 | 0.628240133 | 0.406934471 |
| Hrc | 1.168216595 | 0.474521659 | 1.07734847 | 1.33224598 | 2.069438804 | 1.914931976 |
| Car11 | 1.091342177 | 2.06748232 | 1.230109985 | 2.519025102 | 4.242287403 | 2.788727513 |
| Dbp | 10.24912653 | 7.358710152 | 10.86969914 | 14.13840176 | 34.25220329 | 48.80173763 |
| Klhl25 | 6.291141253 | 2.947944082 | 7.156757771 | 2.524337731 | 2.206997105 | 2.14693335 |
| Cemip | 7.777166626 | 3.099530028 | 8.774128191 | 2.463925327 | 1.751361253 | 2.501630449 |
| Folh1 | 16.54371736 | 0.940290925 | 24.68229677 | 0.923619706 | 0.664861376 | 1.355916939 |
| Nox4 | 11.652078 | 0.422401445 | 26.32824724 | 0.777057064 | 0.935688673 | 1.03087767 |
| Thrsp | 1.861659675 | 2.602743599 | 2.539008005 | 3.988952087 | 7.862792422 | 2.57023662 |
| Scube2 | 0.103537591 | 0.556194137 | 0.286452185 | 0.640912797 | 0.911189824 | 0.683115157 |
| Micalcl | 0.612623387 | 0.704069012 | 0.529100123 | 0.989631776 | 1.658740545 | 1.301485074 |
| Abca15 | 0.606528885 | 0.042498466 | 0.609692447 | 0.031817816 | 0 | 0.051450763 |
| Sez6l2 | 0.238035555 | 0.071736576 | 0.693967357 | 0.016112355 | 0.075084179 | 0.108559847 |
| Slc5a2 | 23.96467072 | 3.529775151 | 32.86470468 | 4.827605333 | 3.298654334 | 5.239906595 |
| Nkx1-2 | 5.102440767 | 9.821512843 | 6.65268038 | 17.53688499 | 8.44944199 | 18.63992282 |
| Cyp2e1 | 29.33550264 | 13.7032646 | 58.44362541 | 7.741830689 | 10.94071156 | 7.164098452 |
| Dhcr7 | 2.867264365 | 2.785764933 | 1.755600734 | 1.812358548 | 1.046929768 | 0.653473149 |
| Slc22a8 | 85.37948811 | 22.66616394 | 104.2064445 | 22.32777558 | 25.30222331 | 18.75967505 |
| Rbp4 | 10.84801083 | 7.216158285 | 15.00631979 | 37.85424757 | 80.52698797 | 7.506696146 |
| Cyp2c23 | 300.1526785 | 364.4607506 | 453.9725199 | 173.3899586 | 112.0426006 | 121.2516216 |
| Gucy2g | 0.215796037 | 0.482101878 | 0.441284458 | 0.108282229 | 0.234460264 | 0.053059689 |
| Metrn | 2.307409174 | 1.796403425 | 1.93448229 | 0.47684025 | 0.607752071 | 0.659035031 |
| Kcnip1 | 2.055182261 | 0.960021264 | 2.188236242 | 0.725284266 | 0.669880572 | 0.563514974 |
| AABR07029467.2 | 13.53680049 | 3.053885823 | 6.383791557 | 17.43034512 | 56.65768684 | 24.93788556 |
| Slc22a5 | 15.06880983 | 6.418463231 | 17.05920191 | 6.38527481 | 5.926171887 | 5.38696448 |
| Gpx3 | 2485.494637 | 458.3214964 | 4020.530477 | 364.1579313 | 237.0767138 | 453.2890528 |
| Kcnj12 | 16.64981574 | 12.31819491 | 19.79169419 | 7.770366619 | 5.836913699 | 6.200538098 |
| Serpinf1 | 27.98404382 | 32.36311287 | 30.13743456 | 17.40594864 | 13.37702354 | 12.07668922 |
| Coro6 | 2.349821467 | 3.939160737 | 1.95732991 | 4.665664014 | 6.780701981 | 4.512933743 |
| Vtn | 0.191033285 | 0.356943555 | 0.563756873 | 0.641368899 | 1.162312359 | 0.900278487 |
| Krt23 | 1.731011453 | 1.783662975 | 1.220751314 | 3.204949195 | 4.92307913 | 1.679526627 |
| Hsd17b1 | 1.409451909 | 0.238930505 | 0.943417471 | 0.286212564 | 0.321090688 | 0.171414038 |
| G6pc | 110.2980999 | 22.17520945 | 101.9399615 | 14.67451385 | 13.55424783 | 8.219559299 |
| Ttyh2 | 2.82745982 | 1.01809297 | 2.314182643 | 0.580940873 | 0.588801378 | 0.58851684 |
| Slc16a5 | 3.126917219 | 4.235349133 | 3.033774997 | 5.707669687 | 9.257503055 | 7.965193664 |
| Smim5 | 2.668969017 | 5.87541633 | 5.233941162 | 7.628189975 | 18.43211606 | 5.378176473 |
| Cbx8 | 1.688928365 | 1.111177376 | 1.44787025 | 0.499150777 | 0.852889182 | 0.672623382 |
| Kcnj15 | 228.0391059 | 79.10461273 | 305.9694873 | 75.72177744 | 69.10889493 | 67.32854437 |
| Polq | 0.284130366 | 0.55743913 | 0.726695634 | 1.240108992 | 1.10310529 | 1.017651489 |
| Fbxo40 | 1.158572539 | 1.923364427 | 1.382705907 | 3.587058248 | 3.578877571 | 2.537284868 |
| Hrg | 10.04358506 | 0.119887093 | 4.84419776 | 0.359029001 | 0.37179748 | 0.854720962 |
| Vom2r60 | 0.099587602 | 0.188985794 | 0.17220247 | 0.391818933 | 0.229927012 | 0.387192604 |
| Flt3 | 0.623292166 | 0.338842562 | 0.495526069 | 1.059840235 | 1.062504681 | 1.367399031 |
| Hspb1 | 7.607362671 | 4.678006189 | 8.201852785 | 23.32749071 | 8.62468639 | 23.69287167 |
| Psph | 21.90350367 | 9.569134342 | 35.74923272 | 7.109124501 | 8.446271468 | 10.76798944 |
| Cux2 | 0.082472205 | 0.30375164 | 0.198918878 | 0.372735843 | 0.63412937 | 0.29897308 |
| LOC498231 | 1.685411919 | 1.421501841 | 0.993034242 | 2.603324493 | 3.458902005 | 2.029827895 |
| Mybph | 0.191352188 | 0.365665318 | 0.368978798 | 1.003811834 | 0.680407408 | 0.442692675 |
| Lmod1 | 2.61243216 | 0.804609932 | 1.767208238 | 2.462296032 | 5.653370258 | 3.368765691 |
| Crb1 | 0.547401872 | 0.147521289 | 0.944925045 | 0.014726195 | 0.030499832 | 0.052917521 |
| F13b | 5.686115465 | 0 | 13.32489825 | 0.230560121 | 0.089534903 | 0.621375886 |
| AABR07021204.1 | 0.721345392 | 0.183128504 | 0.433849912 | 1.349112473 | 0.954111747 | 1.596272422 |
| Colgalt2 | 4.806794792 | 1.609577469 | 6.673190107 | 1.482224904 | 1.534938631 | 1.705055432 |
| Npl | 3.887701285 | 0.627247752 | 5.590240271 | 0.805043652 | 0.861463298 | 1.072771274 |
| Tdrd5 | 6.809073818 | 2.737106796 | 8.099195253 | 2.46878514 | 1.580251245 | 3.466482682 |
| Nphs2 | 67.61798216 | 19.86837608 | 71.72861071 | 20.98789768 | 11.38361363 | 27.60989077 |
| Serpinc1 | 13.13304289 | 3.836942812 | 25.72508295 | 5.03818292 | 3.600283822 | 4.252580608 |
| Slamf9 | 0.393665508 | 0.162255793 | 0.597956103 | 0.728867478 | 1.081864036 | 0.873044256 |
| Apcs | 25.43059473 | 0.68798429 | 39.66100286 | 1.167517207 | 0.853439079 | 0.555272068 |
| Cplx1 | 0.166047202 | 0.246795611 | 0.125289367 | 0.47512623 | 0.606829083 | 0.611793922 |
| Fam69a | 1.867016563 | 2.467640189 | 2.936391576 | 0.880714869 | 1.195082366 | 1.145870586 |
| Dmp1 | 3.794857967 | 2.188130163 | 2.723188616 | 5.474533261 | 9.186728101 | 4.918085207 |
| Slc10a6 | 2.203748529 | 3.138475202 | 1.561426554 | 3.468184311 | 4.788702052 | 6.003359539 |
| Cxcl9 | 0.56030483 | 0.100665702 | 0.198739341 | 0.633079024 | 1.077046949 | 1.096838289 |
| Areg | 0.935107929 | 0.926565977 | 0.757841922 | 1.10001447 | 1.570159463 | 6.596940658 |
| Slc4a4 | 149.638981 | 18.29881852 | 236.8902529 | 22.69759588 | 16.92589518 | 30.77034558 |
| Sult1b1 | 1.369231818 | 4.737767275 | 1.075657185 | 0.95771127 | 0.716279227 | 0.812568466 |
| Zfp518b | 5.205972765 | 2.359078547 | 7.89503257 | 2.175458593 | 2.204482513 | 2.608580476 |
| Pla2g3 | 0.230192839 | 0.298689406 | 0.247668873 | 0.308600066 | 0.61136105 | 0.638841321 |
| Tcn2 | 25.29523128 | 19.38867014 | 45.91802886 | 14.02810819 | 13.38110682 | 13.66045313 |
| Igfbp3 | 24.04997149 | 3.448019869 | 26.57305174 | 3.421619472 | 2.726741402 | 6.31211541 |
| Synpo2l | 0.692929824 | 0.295424874 | 0.568027759 | 0.969341631 | 1.027715581 | 1.56539332 |
| Spetex-2E | 5.525465884 | 7.13912368 | 8.468698814 | 3.919615282 | 2.114660184 | 1.735983015 |
| AABR07016950.1 | 4.06620358 | 2.63807496 | 22.49951709 | 1.185046774 | 1.472630019 | 0.496811023 |
| AABR07016976.1 | 4.014072765 | 3.125104183 | 5.949391539 | 1.804917394 | 0.051919648 | 0.135121679 |
| LOC102546376 | 14.73503403 | 10.95701036 | 63.70517916 | 5.351421748 | 1.368330687 | 0.652868621 |
| Nr1d2 | 6.734031027 | 9.312461833 | 14.13942899 | 10.43237034 | 28.03246541 | 31.59002219 |
| Slc7a7 | 47.08463623 | 2.417419786 | 66.88328437 | 6.172634866 | 5.543816006 | 9.806841346 |
| Myh6 | 0.107178926 | 0.037549242 | 0.074131621 | 0.208031934 | 0.454150498 | 0.085866973 |
| Atp12a | 1.91773877 | 2.171680866 | 2.498775167 | 0.28961296 | 0.260450531 | 0.424497401 |
| Cryl1 | 80.70389261 | 34.97993244 | 95.01640611 | 34.6134513 | 28.61773943 | 27.04115267 |
| Pnma2 | 1.339920206 | 0.771044211 | 2.050539598 | 0.550101063 | 0.443072633 | 0.613158111 |
| Ctsb | 297.187648 | 182.5615794 | 471.6554708 | 144.7841575 | 118.1429186 | 135.7747907 |
| Scel | 0.438466011 | 0.643030771 | 0.423168001 | 0.941453826 | 1.152196635 | 0.884205333 |
| Galnt15 | 0.957520812 | 1.137815586 | 0.697137924 | 1.832970944 | 1.989416199 | 1.789161873 |
| Ogdhl | 3.085889023 | 0.374953338 | 4.688262159 | 0.411723595 | 0.513577347 | 0.924686921 |
| Fcho1 | 0.224615052 | 0.154999411 | 0.150649948 | 0.482033881 | 0.354970236 | 0.307938492 |
| LOC100910404 | 2.295696437 | 2.744758533 | 1.865089693 | 4.989227382 | 5.701146333 | 3.709330163 |
| Cxcl14 | 1.262490628 | 1.09990706 | 1.77415547 | 0.546650609 | 0.740273692 | 0.604415254 |
| Pfn3 | 56.22561825 | 9.574013708 | 64.20869134 | 8.023754199 | 5.517249272 | 12.34043095 |
| Slc34a1 | 302.0130051 | 106.9009383 | 429.5167941 | 94.37795076 | 64.94321702 | 115.0034913 |
| Slc22a23 | 11.19979048 | 2.822110112 | 17.63206361 | 3.538406899 | 3.674791112 | 3.846519287 |
| LOC100910554 | 1.479560081 | 0.863919204 | 1.21286625 | 2.069761083 | 2.589905296 | 2.556653455 |
| Rnf125 | 5.023981022 | 4.222070259 | 4.741449833 | 2.561630178 | 1.734478477 | 1.858708683 |
| Rell2 | 5.563833412 | 2.129607286 | 5.954773307 | 2.225648907 | 1.53653447 | 2.402820463 |
| LOC100911558 | 18.62708497 | 0.391942565 | 105.2358366 | 2.112769106 | 1.640930592 | 1.265347259 |
| Spink1l | 22.58703968 | 1.89120044 | 63.27645633 | 1.520236785 | 1.666908385 | 1.847732516 |
| Tnfaip8 | 55.76271236 | 11.96568128 | 82.5213965 | 13.41988099 | 12.86225082 | 12.59450922 |
| Prdm6 | 0.557270774 | 0.986672332 | 0.397876825 | 0.924165025 | 1.210938392 | 1.6096298 |
| Prelid3a | 1.534736524 | 1.792273762 | 2.191523845 | 3.713873024 | 3.765268579 | 3.289721071 |
| Pdp2 | 41.21931323 | 31.73419661 | 55.09087414 | 24.68246335 | 17.3995598 | 17.51158233 |
| Slc38a7 | 3.787392508 | 1.235234887 | 4.216527756 | 1.431944557 | 1.093616712 | 1.640156683 |
| Herpud1 | 22.86865466 | 31.25273841 | 26.86367877 | 45.07938515 | 45.74893309 | 78.61450706 |
| Ces2g | 27.16198792 | 4.188299698 | 36.46089419 | 4.030343766 | 4.487627863 | 6.800804588 |
| Lcat | 0.924996629 | 1.274101108 | 0.678063029 | 2.48842421 | 1.906922523 | 2.101363679 |
| Dpep3 | 0.32520532 | 0.10549349 | 0.033323297 | 0.682396062 | 1.158144991 | 0.306517635 |
| Dpep2 | 0.870933855 | 2.172855613 | 1.095258355 | 2.409014202 | 3.677514486 | 1.977589677 |
| Maf | 64.83997729 | 19.18226362 | 69.69109206 | 20.91043973 | 17.48568327 | 18.51891825 |
| Hsd17b2 | 3.872371233 | 1.398304559 | 9.985162924 | 1.069158919 | 0.553591201 | 1.368693099 |
| Dpep1 | 10.37434554 | 5.956495567 | 21.18182689 | 4.211767116 | 3.933950724 | 4.599756194 |
| AABR07072671.2 | 0.536606785 | 0.563987122 | 0.247433781 | 0.506696079 | 2.448675496 | 1.087407801 |
| F2rl1 | 1.520175456 | 2.409294005 | 1.782454741 | 3.030373727 | 4.164531693 | 4.042598412 |
| Prlr | 6.586830227 | 14.31803645 | 5.863980804 | 3.13606759 | 3.117694508 | 4.10814764 |
| Agxt2 | 120.5920062 | 87.08858835 | 236.811632 | 60.58311632 | 47.79126613 | 52.47091629 |
| Car3 | 12.52635119 | 1.379583463 | 4.902558711 | 0.085478784 | 0.221296861 | 0.230371388 |
| Fabp5 | 29.31016025 | 36.36785191 | 25.09368687 | 54.23711585 | 85.34125195 | 43.12331461 |
| 1700034I23Rik | 0.489450431 | 1.314640688 | 0.722206722 | 2.208137155 | 1.914419024 | 1.753033182 |
| Postn | 1.240527199 | 0.338807583 | 0.073808677 | 0.041984934 | 0.054347603 | 0.12258174 |
| Vom2r46 | 0.169265808 | 0.304106989 | 0.204130376 | 0.532769517 | 0.452690781 | 0.355894855 |
| AC121415.1 | 2.71761525 | 1.197602283 | 1.607769725 | 3.819612665 | 6.573851571 | 5.509532857 |
| Hmgcs2 | 3.56432789 | 1.499979003 | 3.005754739 | 1.027550995 | 0.331439344 | 0.484253176 |
| Phgdh | 26.89289872 | 6.695413764 | 42.15025544 | 4.605448001 | 8.020102624 | 11.46633228 |
| Mab21l3 | 6.138858003 | 1.136656687 | 7.597948828 | 1.350868977 | 1.157432459 | 1.842014637 |
| Slc16a1 | 13.88527773 | 1.805071234 | 43.66236203 | 2.098681146 | 1.510032966 | 1.726210694 |
| Dram2 | 7.314487083 | 4.661666888 | 9.703363166 | 4.406003906 | 3.133721617 | 2.936001062 |
| Cfi | 1.374955443 | 0.685571465 | 2.20832579 | 0.632138293 | 0.7385452 | 0.422274096 |
| Ddit4l | 6.720909944 | 1.941080211 | 18.36113649 | 1.251059447 | 0.588887284 | 0.34057515 |
| Mttp | 0.501083604 | 0.339775079 | 0.603721093 | 0.135671104 | 0.228306163 | 0.144733788 |
| Stpg2 | 0.031332423 | 0.036590176 | 0.115581095 | 19.09936782 | 0.459571977 | 19.63873254 |
| Ptgfr | 3.767512481 | 3.620342524 | 3.990667431 | 7.318305516 | 13.47301893 | 9.192056344 |
| Col11a2 | 1.439934596 | 4.488158938 | 2.239997926 | 5.2311619 | 6.781246322 | 5.819977664 |
| Hspa1b | 40.95839184 | 76.3859116 | 44.55311203 | 188.574428 | 44.94389668 | 299.8252502 |
| Hspa1l | 0.658437061 | 0.360924648 | 0.570044615 | 1.987861739 | 0.671586263 | 3.723602752 |
| Fkbp5 | 15.76559289 | 10.95981115 | 9.050772676 | 19.73300561 | 37.47068749 | 29.11697321 |
| AABR07044783.1 | 3.117026429 | 3.805538835 | 3.397222407 | 5.946024164 | 10.69863556 | 5.2748817 |
| Cdk1 | 1.190498851 | 0.802079488 | 0.506721938 | 0.384321335 | 0.348240645 | 0.431572196 |
| Naglt1 | 19.93104519 | 6.952633051 | 29.47470236 | 4.755466425 | 5.257331981 | 7.204849865 |
| Slc16a10 | 2.264337536 | 1.911373244 | 2.632959362 | 0.903795673 | 1.069643645 | 0.893124274 |
| Creb3l1 | 8.07593211 | 5.987330961 | 9.794928661 | 5.032498633 | 2.725388194 | 3.840526642 |
| Slc5a12 | 30.37094373 | 0.408322607 | 45.47475809 | 1.895362243 | 0.654256251 | 3.075866683 |
| Capn3 | 0.103482312 | 0.17120022 | 0.135196867 | 0.18095215 | 0.271711901 | 0.349503201 |
| Gatm | 212.4660177 | 243.9704871 | 390.04091 | 106.8118096 | 130.1862995 | 83.53769547 |
| Gfra4 | 1.634414832 | 4.303883206 | 1.211738212 | 5.514229552 | 4.665763297 | 4.772512014 |
| Hao1 | 0.690988844 | 0.453904074 | 0.398275766 | 1.495250194 | 0.844596628 | 0.936217411 |
| Tpx2 | 6.093188815 | 2.203559054 | 2.220505531 | 1.412610365 | 1.590981027 | 1.852596192 |
| Spata25 | 4.836376273 | 0.905577974 | 2.615889318 | 0.963458748 | 0.931208243 | 1.134805867 |
| Neurl2 | 13.77789517 | 5.285571616 | 19.74473991 | 4.888317943 | 4.82111018 | 5.290657184 |
| Ctsa | 133.1067575 | 53.82313227 | 224.826283 | 51.69104996 | 44.09669063 | 56.33981705 |
| Slc13a3 | 99.23664934 | 51.9523244 | 128.6935081 | 39.90703429 | 29.15469431 | 29.45632874 |
| Cyp24a1 | 12.25348528 | 0.352455359 | 21.66367134 | 1.139947828 | 0.470009446 | 1.649906822 |
| Pck1 | 1398.969593 | 320.7227806 | 1556.146414 | 219.3756237 | 336.5145942 | 272.7024599 |
| Edn3 | 0.507920259 | 2.787814749 | 1.295940913 | 3.836868423 | 7.063684451 | 2.505369335 |
| RGD1563349 | 0.865479154 | 0.656306053 | 0.518285095 | 0.259440303 | 0.158757783 | 0.2436641 |
| Pdk4 | 20.26940876 | 19.06608721 | 20.54880139 | 7.670484573 | 5.600456587 | 6.155295014 |
| Slc13a1 | 39.36471233 | 10.33599552 | 52.6576536 | 10.33945303 | 9.236856205 | 15.1311326 |
| Grm8 | 0.394733839 | 0.075260792 | 0.312025722 | 0.033807818 | 0.087525392 | 0.083521597 |
| Akr1b8 | 21.43127615 | 5.387030174 | 35.28645948 | 5.909222316 | 5.224897661 | 8.658792536 |
| LOC100910708 | 3.81969762 | 1.231992889 | 6.240012468 | 1.030510269 | 0.830012753 | 1.851532201 |
| Fam180a | 2.671876448 | 2.340174019 | 2.686497002 | 1.732577862 | 0.685422856 | 0.874423275 |
| AABR07060287.1 | 0.734175646 | 1.071717952 | 0.846336002 | 2.182461142 | 2.060659367 | 1.44163913 |
| Tmem178b | 0.101149343 | 0.350430853 | 0.105719124 | 0.233471902 | 0.586121401 | 0.572021221 |
| Clec5a | 0.21092339 | 0.293686246 | 0.299256929 | 0.655376697 | 0.546473202 | 0.611701023 |
| Inmt | 21.86624218 | 38.16708757 | 6.883125433 | 7.830720632 | 9.502982522 | 4.808933415 |
| Abcg2 | 9.606356563 | 9.37612604 | 23.48323323 | 7.657889722 | 5.691861713 | 4.005655266 |
| Ptcd3 | 75.47973447 | 27.76379919 | 61.89205397 | 28.40975369 | 22.02989539 | 30.0301911 |
| Retsat | 63.5175875 | 83.62851896 | 97.025208 | 23.58589585 | 52.19655289 | 21.94186429 |
| Slc4a5 | 2.50349579 | 2.036435951 | 2.046043294 | 1.313305444 | 0.9660773 | 0.593976896 |
| Gxylt2 | 1.427655414 | 0.904257005 | 1.026507306 | 0.406200302 | 0.315484783 | 0.798246925 |
| Lrtm2 | 0.223853129 | 0.442397858 | 0.309661531 | 0.686518568 | 0.346112537 | 0.86018826 |
| Cd27 | 0.081989158 | 0.335115867 | 0.283544041 | 0.623653423 | 0.712643292 | 0.946651969 |
| Pzp | 8.796384847 | 0.616989027 | 30.01249837 | 1.094230105 | 0.864768718 | 1.241695908 |
| LOC102551184 | 11.38913503 | 5.924676681 | 6.01549589 | 15.31672955 | 14.2865565 | 18.93204863 |
| Kcnb2 | 0.238954995 | 0.348816199 | 0.385644366 | 0.814794989 | 0.713961171 | 0.50675315 |
| RGD1559441 | 0.254623871 | 0.203450792 | 0.173024012 | 0.492110276 | 0.429529816 | 0.37893489 |
| LOC100912373 | 1.197820753 | 0.174852709 | 3.728193447 | 0.196363486 | 0.48803266 | 0.211685412 |
| Cd72 | 0.592125369 | 0.998814939 | 1.880899888 | 2.692059413 | 3.097555108 | 2.60446349 |
| Spag8 | 2.670922799 | 2.002136778 | 1.531158943 | 4.411684186 | 4.666619186 | 4.966841441 |
| Baat | 6.145864953 | 0.996829851 | 5.020566544 | 1.273699128 | 0.86559169 | 1.001206947 |
| Bnc2 | 12.78948976 | 4.222057811 | 17.98958741 | 3.823948208 | 3.266753613 | 4.561928753 |
| Fam151a | 24.02782087 | 3.161670028 | 46.40307152 | 3.599043483 | 3.710326395 | 4.349631203 |
| Calr4 | 1.436244486 | 0.109983883 | 1.107392413 | 0.07410861 | 0.102325604 | 0.177535967 |
| Catsper4 | 0.123566174 | 0.309216983 | 0.309305748 | 0.611173303 | 0.939774002 | 0.349396605 |
| Slc30a2 | 7.437807941 | 0.610823703 | 8.933449238 | 0.856088842 | 0.738778931 | 0.85781263 |
| Hspb7 | 0.579727928 | 1.203572384 | 0.638591312 | 1.233370217 | 2.379503872 | 1.502639294 |
| Car6 | 9.068537584 | 1.097439183 | 12.28473633 | 1.207799672 | 1.684688741 | 2.39150632 |
| Tnfrsf9 | 1.736962605 | 1.080361849 | 2.402783029 | 1.049850321 | 0.533337075 | 0.854164865 |
| Per3 | 1.823516994 | 2.333713079 | 2.934872312 | 4.371514023 | 17.13631031 | 14.84229243 |
| Tp73 | 0.3605175 | 0.063549332 | 0.263470867 | 0.04995712 | 0 | 0.064113326 |
| Nrxn1 | 1.554831155 | 0.777345723 | 2.189775647 | 0.510756435 | 0.188900334 | 0.739765048 |
| Plb1 | 0.38768351 | 1.097891922 | 0.420095494 | 0.284724109 | 0.200076796 | 0.274054171 |
| Greb1 | 5.108690087 | 2.293103767 | 4.926166002 | 1.597501045 | 1.85771376 | 1.607655061 |
| Plekhh1 | 6.926465189 | 9.985380195 | 10.10815847 | 14.90764901 | 23.34205351 | 19.59231263 |
| Smoc1 | 3.564290595 | 1.927847578 | 5.968585602 | 1.692648691 | 1.100624178 | 1.414514212 |
| Acot5 | 1.43918919 | 0.283490396 | 0.911480705 | 0.1637311 | 0.094196678 | 0.294177956 |
| Tmem63c | 0.245330329 | 0.373113816 | 0.152585622 | 0.407043265 | 0.892629146 | 0.419445185 |
| Kcnk10 | 0.359282554 | 0.259147501 | 0.292355518 | 0.521079448 | 0.952930198 | 0.647393588 |
| AABR07065589.1 | 1.881770589 | 1.587113706 | 0.964110915 | 3.948621872 | 1.93094044 | 3.744342718 |
| Ikzf4 | 1.88942875 | 1.156798117 | 1.759379237 | 1.03928804 | 0.478332976 | 0.674303985 |
| Adamtsl5 | 0.799140761 | 0.610196025 | 0.935398573 | 1.322155896 | 1.870095336 | 1.622317252 |
| Angptl4 | 2.258523387 | 2.078042945 | 1.483239705 | 0.753960903 | 0.204488614 | 0.145141295 |
| LOC362863 | 0.876509616 | 0.461007424 | 0.549171795 | 1.22147199 | 1.526618319 | 1.002721526 |
| Pah | 116.3606551 | 7.496290011 | 273.4780873 | 11.82368968 | 25.2536345 | 24.36698749 |
| Acss3 | 21.98346306 | 8.203837126 | 33.16300729 | 7.844078166 | 10.04805257 | 8.897560003 |
| Agap2 | 0.423308481 | 0.517514255 | 0.463578639 | 0.860493423 | 1.121056185 | 0.779264394 |
| LOC100365958 | 11.05744354 | 0.85334913 | 13.63038038 | 1.244020933 | 0.644131597 | 1.325495808 |
| Sdr9c7 | 0.402538672 | 0.235043439 | 0.208815612 | 0.63350164 | 0.792704658 | 0.474258854 |
| Enpp2 | 4.872357231 | 0.768145356 | 5.773977381 | 1.188532206 | 1.27050433 | 0.975878796 |
| Fer1l6 | 0.181296435 | 0.230966155 | 0.273591067 | 0.455356238 | 0.513333446 | 0.481566312 |
| Ndrg1 | 412.3946405 | 267.2496032 | 463.6151784 | 178.7972329 | 145.5926201 | 157.4617351 |
| Tef | 10.67737937 | 12.3331747 | 9.725199628 | 15.87558457 | 26.6330958 | 26.84170685 |
| Fbln1 | 2.336780108 | 1.300036133 | 1.350353921 | 0.904918052 | 0.697377105 | 0.746769964 |
| Creld2 | 24.86568665 | 24.34380975 | 18.44734997 | 44.07014109 | 35.04537558 | 60.39569883 |
| Prph | 1.43735928 | 1.119037387 | 1.206596084 | 2.571409537 | 3.103331847 | 1.667399982 |
| Fat3 | 2.094022783 | 0.65689379 | 3.241092884 | 0.972036611 | 0.788336875 | 0.414341515 |
| Rgl3 | 9.684746988 | 13.07913973 | 10.33512955 | 18.54682606 | 24.86758849 | 21.67952278 |
| Barx2 | 6.965356667 | 4.809996087 | 6.304242752 | 2.579256677 | 1.077765939 | 0.569110625 |
| AABR07070043.1 | 11.66750698 | 6.080484504 | 6.838109212 | 3.374933209 | 3.465340766 | 4.136737861 |
| Nectin1 | 5.421128191 | 1.179341949 | 6.753547032 | 1.332552596 | 1.090491849 | 1.950410002 |
| Usp2 | 5.949354376 | 6.755518679 | 4.58772481 | 10.53159241 | 13.98681141 | 17.15649988 |
| Tmprss13 | 3.938470572 | 0.398370424 | 6.539726305 | 0.574814048 | 0.314475232 | 0.565104086 |
| Zbtb16 | 21.41275714 | 38.4430333 | 9.396941171 | 50.08319165 | 75.704029 | 52.40435673 |
| Cib2 | 2.661386718 | 4.260078861 | 3.258393607 | 1.85348722 | 1.221439221 | 1.27585063 |
| Cyp1a1 | 4.763514164 | 9.581272248 | 2.168262788 | 8.556594743 | 24.17455967 | 355.6046101 |
| Stra6 | 8.173232497 | 0.137730821 | 13.51962281 | 0.692943013 | 0.525376037 | 0.733672733 |
| Slc24a1 | 0.071189773 | 0.20464199 | 0.176756392 | 0.270035578 | 0.773469855 | 0.29420289 |
| Cilp | 2.261574012 | 0.01827736 | 3.247568379 | 0.098524238 | 0.051014073 | 0.191771531 |
| Ctsh | 367.8278589 | 122.3356877 | 694.144523 | 183.6640081 | 105.7732883 | 125.4176728 |
| Clstn2 | 5.184695147 | 0.884707293 | 8.482091676 | 1.043223294 | 0.835965312 | 1.138012708 |
| Acpp | 8.130171315 | 8.747703635 | 3.385583339 | 3.695930078 | 2.003632259 | 2.130996411 |
| Dock3 | 2.208673526 | 1.924240506 | 3.487166757 | 1.140253843 | 0.995794702 | 1.047957657 |
| Camkv | 0.196592538 | 0.229581849 | 0.148337065 | 0.337517056 | 0.378647193 | 0.901209895 |
| Bsn | 0.122797248 | 0.075227962 | 0.116958604 | 0.038017204 | 0.061241048 | 0.037947801 |
| LOC100912849 | 1.248453273 | 0.546731477 | 1.48715273 | 2.89258277 | 2.769382325 | 3.186927854 |
| LOC102548541 | 0.983607116 | 0.906838267 | 0.57290437 | 1.683753958 | 1.743634849 | 1.854169711 |
| Npas2 | 4.052140989 | 3.58439869 | 4.601469224 | 2.022594459 | 0.44354665 | 0.484536497 |
| Il1rl1 | 0.518971962 | 0.435936682 | 0.394638749 | 0.171945344 | 0.197844882 | 0.257447168 |
| Il18rap | 0.237482641 | 0.13511121 | 0.14600692 | 0.33860394 | 0.218326769 | 0.48784742 |
| Dnah7 | 1.796592793 | 0.074809661 | 3.19822548 | 0.201630998 | 0.132873864 | 0.175647627 |
| Icos | 0.381660308 | 0.552674211 | 0.774341446 | 0.160171728 | 0.199041676 | 0.259004505 |
| Idh1 | 120.6378776 | 62.42048265 | 127.5325361 | 42.9889825 | 47.32025155 | 53.93835596 |
| Cfap65 | 0.834498597 | 1.84152954 | 0.679360679 | 3.109665764 | 2.319836106 | 1.366960528 |
| Dnajb3 | 1.465455136 | 1.190515879 | 1.11642903 | 1.738068602 | 1.869107332 | 3.933542222 |
| Spp2 | 65.56460439 | 1.30405582 | 100.1861213 | 2.092119613 | 3.46643774 | 3.458225302 |
| Per2 | 13.51248817 | 8.532527975 | 7.835405926 | 24.96065475 | 31.01316887 | 32.90616724 |
| Lama1 | 1.690062614 | 0.949357572 | 3.54279293 | 1.064546464 | 0.939701423 | 0.74030147 |
| Epb41l3 | 29.83803941 | 3.515166892 | 55.30594356 | 4.952151648 | 5.510976256 | 6.627927325 |
| Akain1 | 0.914139925 | 0.144261843 | 0.831642444 | 0.12960741 | 0.053686703 | 0.163007211 |
| Cacna1f | 0.06941297 | 0.099767198 | 0.098482735 | 0.145653022 | 0.2494546 | 0.186207352 |
| Nhs | 4.346954568 | 2.025298475 | 4.881221131 | 1.600980221 | 1.529444052 | 2.080423221 |
| Slc16a2 | 7.422603859 | 0.92831675 | 12.49005542 | 1.052945589 | 0.906861057 | 0.814803261 |
| Slc6a8 | 55.07142264 | 45.61424274 | 68.16333102 | 28.25803951 | 24.01090153 | 26.83198186 |
| AABR07000658.2 | 1.393152709 | 1.398144175 | 0.341271876 | 0.319739035 | 0.28380875 | 0.49241108 |
| AABR07000986.1 | 0.122236331 | 0.337404978 | 0.07173622 | 0.454695965 | 0.422572712 | 0.408479298 |
| AABR07002627.1 | 2.24787477 | 2.094165369 | 3.726789963 | 1.695941841 | 1.125101616 | 1.047448327 |
| LOC361559 | 0.061801661 | 0.07217232 | 0.085491756 | 0.285299954 | 0.376022613 | 0.069900329 |
| AABR07004221.2 | 10.44647935 | 2.189645966 | 9.930342421 | 2.192041744 | 1.105897115 | 2.423675098 |
| AABR07004221.1 | 7.695625058 | 1.462998911 | 5.281511759 | 0.563307552 | 0.486117481 | 0.590393155 |
| AABR07071947.2 | 4.455686682 | 2.081350175 | 3.287285767 | 7.713428588 | 4.599006622 | 7.34937686 |
| AABR07005921.1 | 0.229002949 | 0.342645846 | 0.277187228 | 0.465512836 | 0.839731872 | 0.465412466 |
| AC099294.7 | 53.07041101 | 15.86823025 | 34.28332503 | 83.92388392 | 97.77211446 | 84.3345161 |
| Rn60_1_2432.3 | 0.108766762 | 0 | 0.200612978 | 0.228231231 | 13.29457656 | 7.483708905 |
| AABR07029907.1 | 986.1866811 | 250.0593568 | 760.0967813 | 1907.170262 | 2194.106528 | 1487.138908 |
| AABR07034310.1 | 61.40906793 | 39.59202004 | 36.87008325 | 13.75827571 | 30.58015867 | 16.88173695 |
| AABR07034442.1 | 15.58711372 | 7.650417383 | 9.583127651 | 18.01271096 | 26.99821701 | 26.18903822 |
| AABR07035758.1 | 0.77271377 | 0.751982829 | 0.593841075 | 1.486308499 | 1.795695364 | 1.375697311 |
| AC097575.2 | 18.78656548 | 3.478142802 | 8.240071371 | 59.37168383 | 51.27746465 | 28.93584446 |
| AC097575.3 | 9.164178281 | 3.478142802 | 7.394935846 | 24.51786134 | 23.64737447 | 12.09259171 |
| AABR07036645.1 | 23.07409174 | 7.091066151 | 19.4873637 | 30.83438245 | 32.45875865 | 36.17061737 |
| AABR07020664.1 | 5.943886033 | 1.810774652 | 2.621610399 | 12.20124158 | 9.827350992 | 9.499594547 |
| Rn5-8s | 12.6681288 | 22.68399619 | 18.69240926 | 21.48729909 | 35.78587196 | 54.32774659 |
| AABR07017046.1 | 1.852543567 | 1.081705288 | 2.349113261 | 4.373204869 | 4.780329464 | 4.583482343 |
| AABR07024873.1 | 9.014993984 | 4.386566503 | 3.464072938 | 13.92475891 | 11.97130243 | 13.92556346 |
| AABR07025301.1 | 7.627269215 | 5.23950999 | 2.20674276 | 2.196725594 | 2.274849767 | 1.550562975 |
| AABR07026565.1 | 0.937384977 | 1.094683337 | 1.196960917 | 3.328711742 | 1.018459526 | 2.718519502 |
| AABR07026778.1 | 4.597069047 | 1.088205921 | 7.390466379 | 1.042841161 | 0.573712111 | 1.171054555 |
| AABR07028905.1 | 52.57273452 | 22.68399619 | 41.27907046 | 107.1042176 | 121.4655077 | 102.386907 |
| AABR07031489.1 | 0.340238216 | 0.286962045 | 0.087159237 | 0.674277053 | 1.150070333 | 1.193667077 |
| AABR07032277.3 | 5.084846144 | 5.23950999 | 3.31011414 | 3.295088391 | 1.137424883 | 1.409602705 |
| AABR07007690.1 | 6.339984087 | 4.583347056 | 2.50578734 | 10.76952788 | 7.872377297 | 10.81310187 |
| AABR07007705.1 | 2.106764898 | 1.640194432 | 0.863508037 | 9.33267271 | 11.19056531 | 3.971402403 |
| AABR07010863.1 | 67.19175516 | 150.8978877 | 67.32772162 | 33.21449098 | 37.20354304 | 18.26845105 |
| AABR07012588.2 | 8.026987067 | 6.630361732 | 7.944273937 | 15.40560806 | 37.86295404 | 10.33366928 |
| AABR07013566.1 | 0.142307174 | 0.214658339 | 0.098428504 | 9.592875328 | 0.244807621 | 11.6525087 |
| AABR07013582.1 | 0.301904004 | 0 | 0 | 13.93703608 | 0 | 14.79687624 |
| AABR07013746.1 | 1.254842995 | 0.376254576 | 2.580325196 | 0.462572589 | 0.368479602 | 0.255726349 |
| Rn60_20_0062.1 | 0.604514216 | 1.213360208 | 0.993034242 | 0.535142175 | 0.472074087 | 0.195860586 |
| AABR07045373.3 | 16.60472023 | 5.641042531 | 10.7192014 | 23.43956068 | 29.84943058 | 26.03681108 |
| AABR07045373.2 | 16.52748897 | 5.614805124 | 10.66934465 | 23.33053946 | 29.71059602 | 25.91570963 |
| AABR07051515.1 | 12.58753633 | 7.649892915 | 19.54480914 | 5.862094201 | 4.954145984 | 6.17422799 |
| AABR07054460.7 | 5.139229525 | 0 | 3.611033608 | 14.89208779 | 22.06899709 | 16.37702051 |
| AABR07059563.2 | 284.7127894 | 83.29130719 | 227.1165412 | 482.0243589 | 658.1758882 | 420.9164618 |
| AABR07059563.1 | 1244.214573 | 364.4670755 | 985.9869024 | 2133.030675 | 2917.636349 | 1847.961863 |
| AABR07060727.1 | 8.748926453 | 0.392963249 | 2.792908806 | 16.2400785 | 22.30165218 | 13.00358495 |
| Mir207 | 13.25281167 | 15.47670643 | 8.784533681 | 4.779688655 | 3.599762268 | 5.074569737 |
| Rn50_5_0814.26 | 2.24561531 | 3.697213203 | 1.833293985 | 1.042841161 | 1.09992736 | 1.197077989 |
| Rn50_5_0814.25 | 1.384905606 | 2.482368043 | 1.455394153 | 0.743401227 | 0.664861376 | 1.062472228 |
| AABR07048790.1 | 42.43823148 | 70.76420551 | 51.20941288 | 62.46823034 | 123.6447756 | 147.2620237 |
| AABR07049316.1 | 9.691118532 | 9.259643109 | 9.749790741 | 17.56239319 | 32.86419325 | 25.46511359 |
| AABR07049565.1 | 10.08059611 | 1.605296678 | 5.070813151 | 19.22969517 | 22.90061528 | 13.38822654 |
| AABR07050498.1 | 0.447833574 | 0.505549762 | 0.481832696 | 0.093971362 | 0.162188919 | 0.056279886 |
| LOC100362830 | 55.23606809 | 12.23148407 | 10.77764126 | 6.593389935 | 9.103835634 | 9.456365448 |
| AABR07050652.1 | 4.272521724 | 1.179943268 | 6.788839993 | 1.120658174 | 1.097782729 | 1.03396059 |
| AABR07063419.1 | 47.92775396 | 64.84664128 | 31.93286583 | 103.8921987 | 112.1749448 | 93.33228261 |
| AABR07064316.3 | 10.83356747 | 6.440763499 | 8.719325053 | 14.46624172 | 19.90295388 | 16.52329414 |
| AC128402.2 | 13.01792042 | 2.252207279 | 10.67141275 | 26.30450093 | 28.28768409 | 19.54088048 |
| AABR07065438.1 | 48.1619224 | 37.31293223 | 25.67444895 | 69.63334844 | 80.53322696 | 74.51313671 |
| AC120292.1 | 14.13288119 | 14.73612185 | 9.775180821 | 29.12622774 | 23.03285389 | 32.82612298 |
| LOC102549714 | 5.428241089 | 3.989662131 | 1.050212477 | 0.916009128 | 0.577400176 | 0.6440112 |
| AABR07057377.1 | 1.030325297 | 0.566220967 | 0.503037984 | 2.034812022 | 2.304725842 | 2.913355233 |
| AC091481.1 | 2.542006486 | 0.183893665 | 3.360826196 | 0.424834317 | 0.097765131 | 0.296841127 |
| AC091481.2 | 25.25730609 | 1.282418309 | 27.76557499 | 3.168405511 | 0.894841754 | 1.897572819 |
| AABR07058870.2 | 164.2762776 | 48.30572625 | 102.2704168 | 249.2326787 | 384.1484109 | 226.1277783 |
| LOC100362400 | 20.64353433 | 7.311765626 | 8.78667939 | 6.759426908 | 5.126627725 | 5.773035796 |
| AC105648.9 | 6.800784935 | 4.254639691 | 5.151831783 | 12.23182114 | 18.47246427 | 10.53068607 |
| AABR07069840.1 | 0.361159697 | 0.210882141 | 0.370074873 | 0.484176253 | 1.002790918 | 0.71863472 |
| AABR07070032.2 | 97.29575352 | 107.3351046 | 61.70998505 | 33.48885752 | 24.23411384 | 20.29827895 |
| AABR07071445.1 | 16.51895204 | 18.43354878 | 21.66620165 | 8.473084434 | 3.190698374 | 6.22788104 |
| AABR07071745.1 | 12.57140171 | 5.946714786 | 6.163660813 | 18.53226349 | 14.17738883 | 18.44843579 |
| AABR07067449.1 | 5.337137742 | 6.23273884 | 3.626733754 | 17.9776748 | 18.00663691 | 9.796125926 |
| AY172581.21 | 65.06243897 | 79.16825798 | 55.3861352 | 144.1617746 | 158.6810698 | 87.91156023 |
| AABR07041709.1 | 1.76241233 | 3.689619011 | 2.021746361 | 4.73545637 | 6.421731591 | 3.666655179 |

**Table S3.** List of strong co-expressed lncRNA-PCG pairs by WGCNA

| lncRNA | target_gene | weight_value | distance |
| --- | --- | --- | --- |
| AABR07051515.1 | Ggta1 | 0.813846194 | 50544 |
| TCONS_00030558 | Scgb3a2 | 0.941300524 | 97456 |
| TCONS_00007296 | Abca15 | 0.91985897 | 11616 |
| TCONS_00051715 | Slc46a2 | 0.825049166 | 201157 |
| TCONS_00038977 | Lpar3 | 0.906862488 | 25918 |
| TCONS_00002134 | Cemip | 0.93084533 | 154097 |
| TCONS_00002134 | Arnt2 | 0.876974816 | 175481 |
| TCONS_00002282 | Tsku | 0.911507596 | 1700 |
| TCONS_00002171 | Olr34 | 0.842872132 | 7802 |
| TCONS_00002171 | Folh1 | 0.996780726 | 41001 |
| TCONS_00002284 | Tsku | 0.862599075 | 4005 |
| TCONS_00023309 | Spetex-2E | 0.87440272 | 22312 |
| TCONS_00004582 | Hspa12a | 0.991403913 | 32089 |
| TCONS_00055762 | Nrxn1 | 0.982756411 | 653531 |
| TCONS_00067183 | LOC100361838 | 0.876310262 | 31425 |
| TCONS_00020239 | Ildr2 | 0.941650354 | 32584 |
| TCONS_00022603 | Adgra3 | 0.903428756 | 35878 |
| TCONS_00023471 | Acox2 | 0.851600274 | 93592 |
| TCONS_00021009 | Slc4a4 | 0.976981795 | 220558 |
| TCONS_00067182 | LOC100361838 | 0.854850195 | 33154 |
| TCONS_00021914 | Sptbn1 | 0.851242955 | 107457 |
| TCONS_00036540 | Rpe65 | 0.843307971 | 30803 |
| TCONS_00066106 | Stra6 | 0.997115143 | 11374 |
| TCONS_00051873 | Bnc2 | 0.825827409 | 188535 |
| TCONS_00063420 | Fat3 | 0.972065599 | 231256 |
| TCONS_00029905 | Dhtkd1 | 0.821232916 | 5563 |

**Table S4.** List of significantly differentially expressed circRNAs

| ID | Model_1 | Model_2 | Model_3 | Control_1 | Control_2 | Control_3 |
| --- | --- | --- | --- | --- | --- | --- |
| 9:20596862-20599410 | 3.430111946 | 2.67307614 | 2.663441868 | 0.878822852 | 1.915943421 | 0.712793164 |
| 1:29249554-29295801 | 0.37419403 | 0.70344109 | 0.302663849 | 1.004368974 | 1.297897156 | 1.544385188 |
| X:33925153-33931243 | 0.686022389 | 0.633096981 | 0.665860467 | 0 | 0 | 0 |
| 3:123819851-123833222 | 0.748388061 | 1.195849852 | 0 | 0 | 0 | 0 |
| 2:238543064-238577394 | 1.184947763 | 0 | 0.726393237 | 0 | 0 | 0 |
| 11:34879057-34932316 | 0 | 1.055161634 | 0.847458776 | 0 | 0 | 0 |

**Scanned copy of H & E staining with high-resolution**

Ctrl:


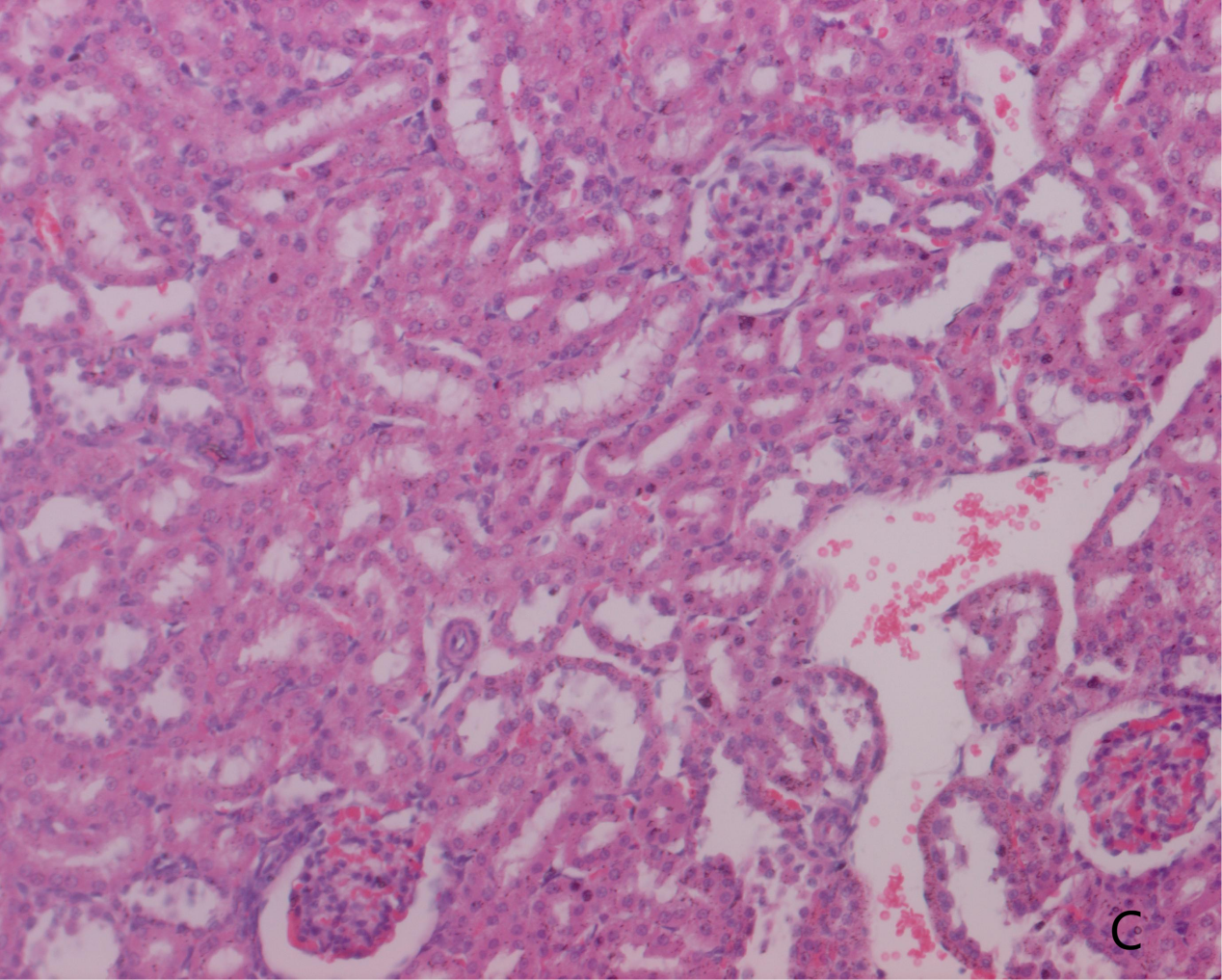


**Scanned copy of H & E staining with high-resolution**

1. TPL:


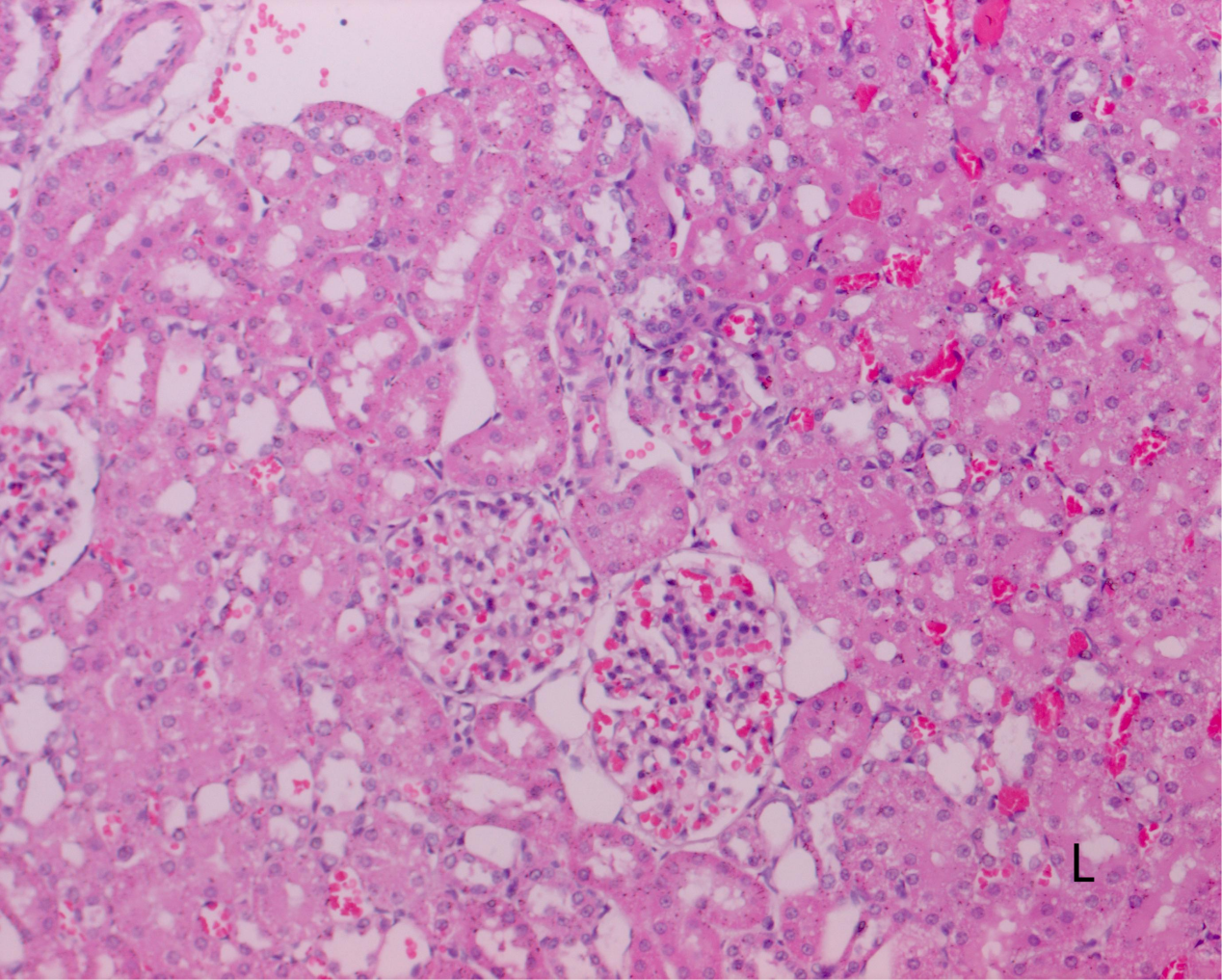


**Scanned copy of H & E staining with high-resolution**

1. TPL：


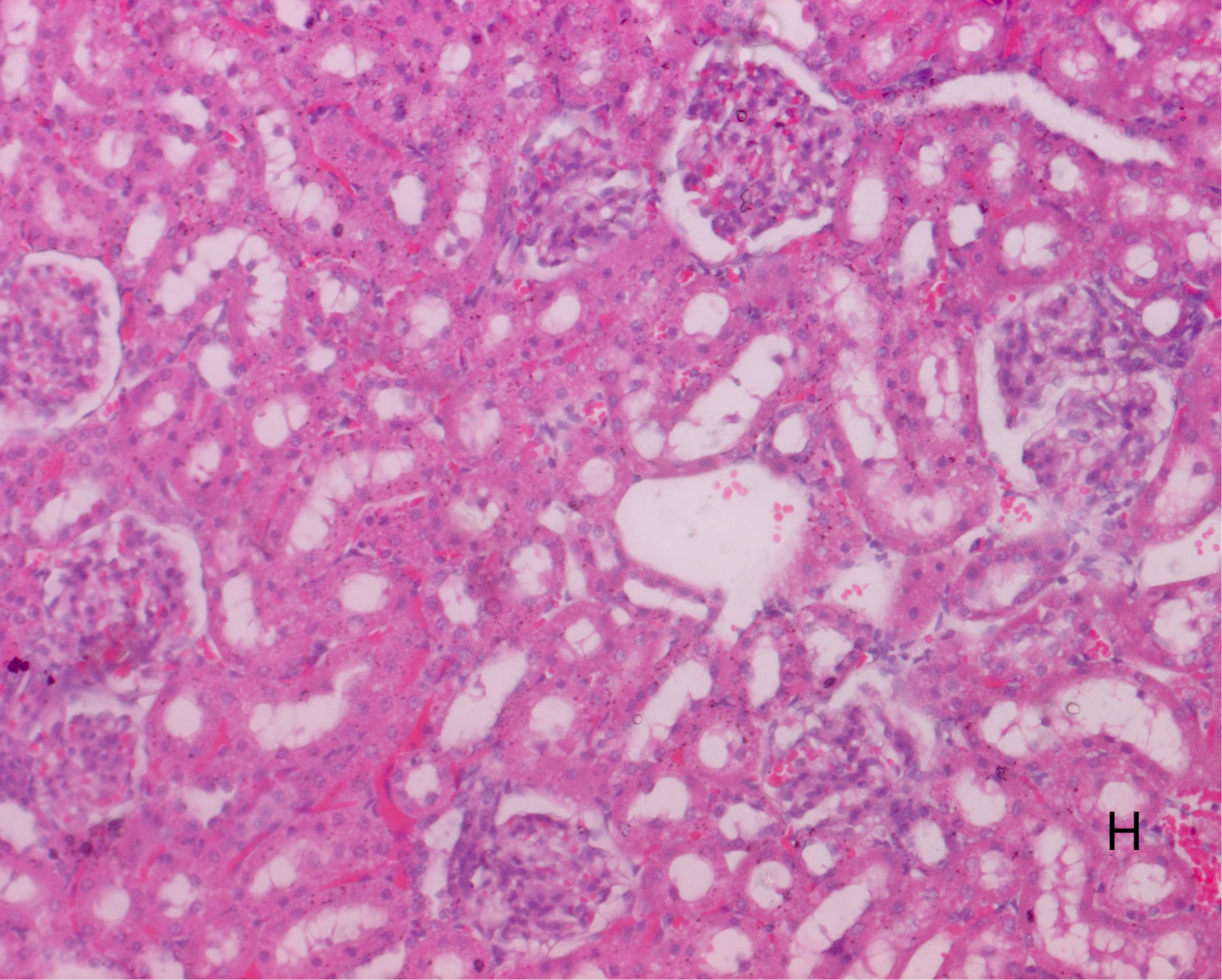


**Raw pictures of western-blot gels see below:**

1. **Figure 2e, c-Jun**

**
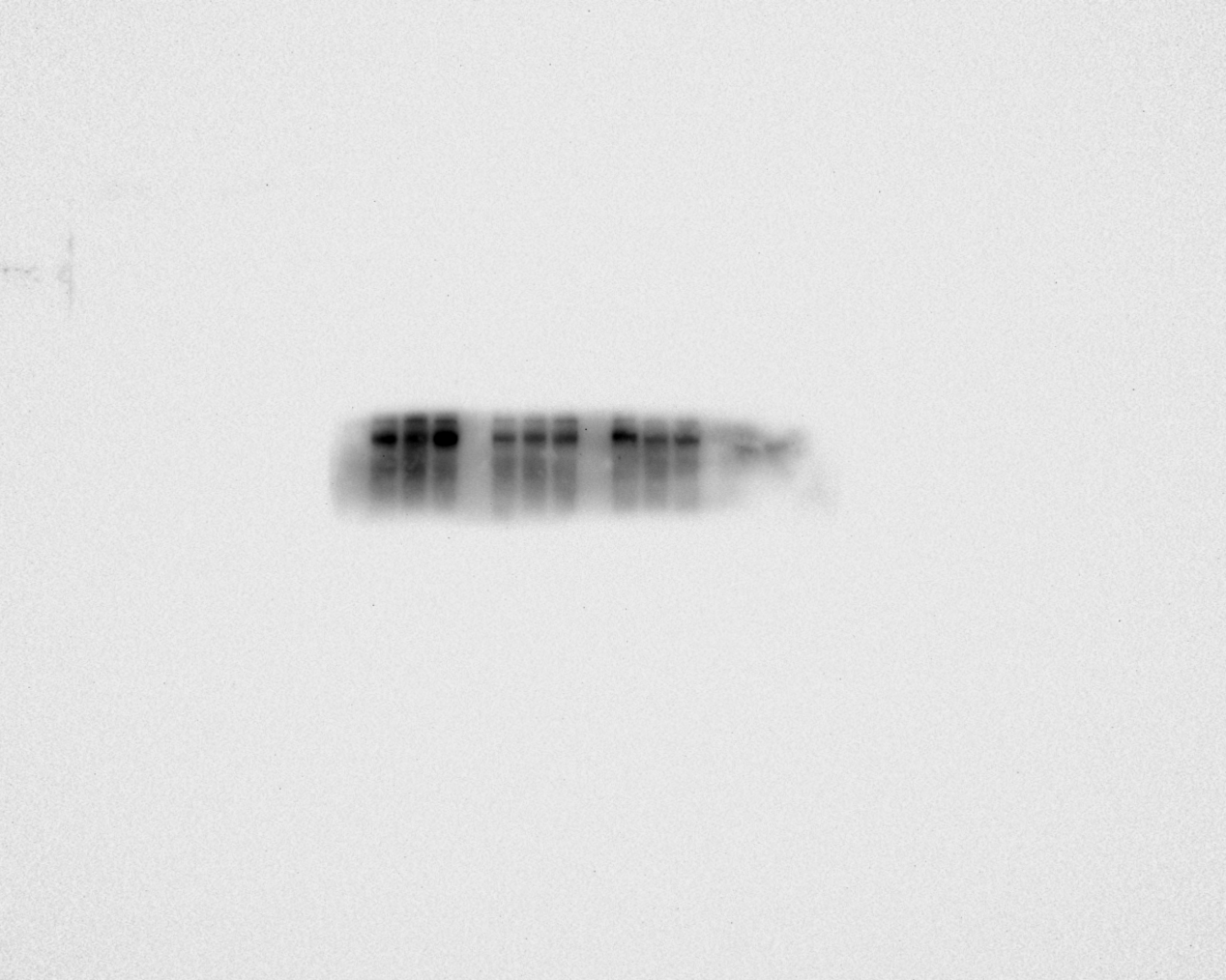
**

**Figure 2e, pc-Jun (1)**

**
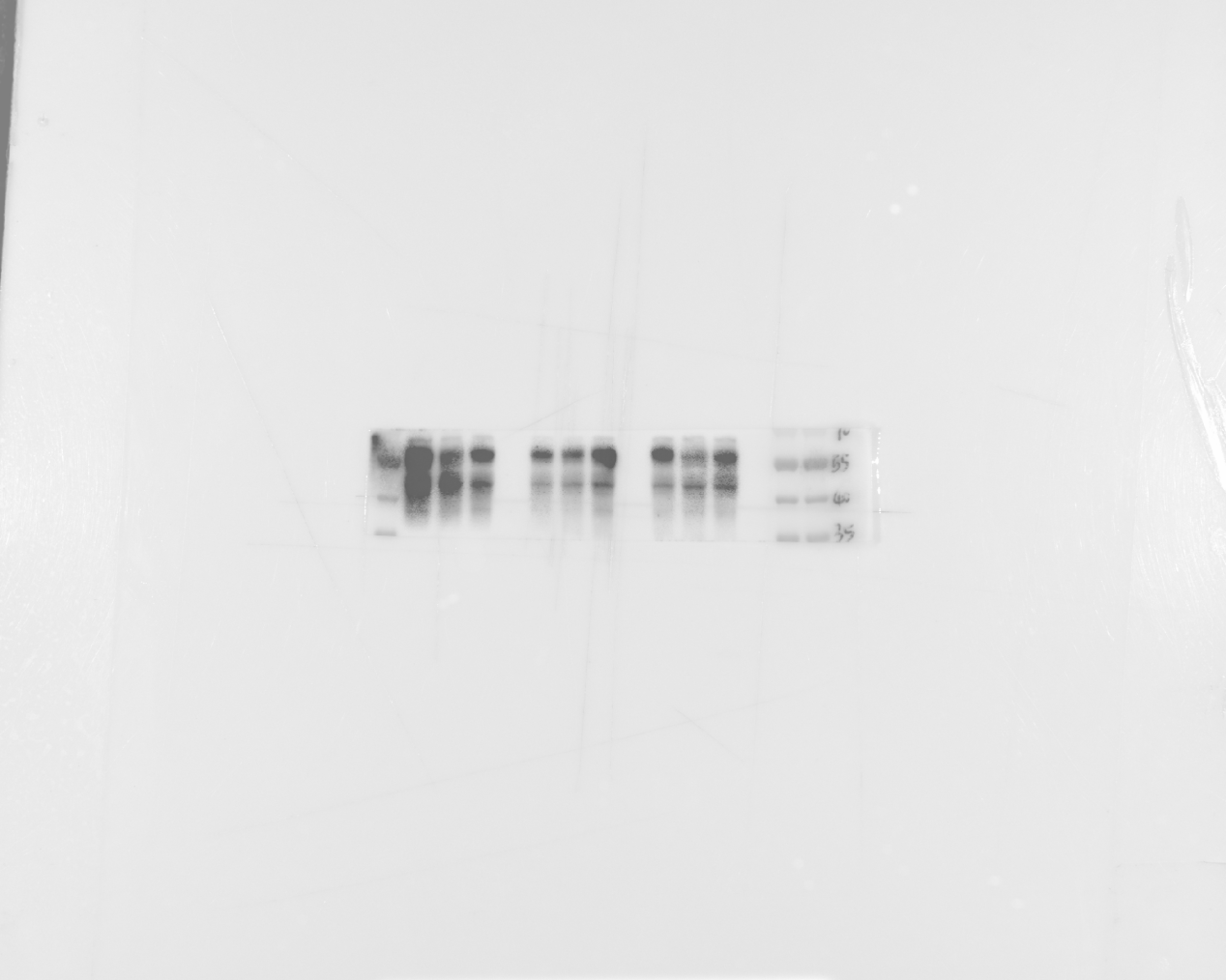
**

**Figure 2e, pc-Jun (1)**

**
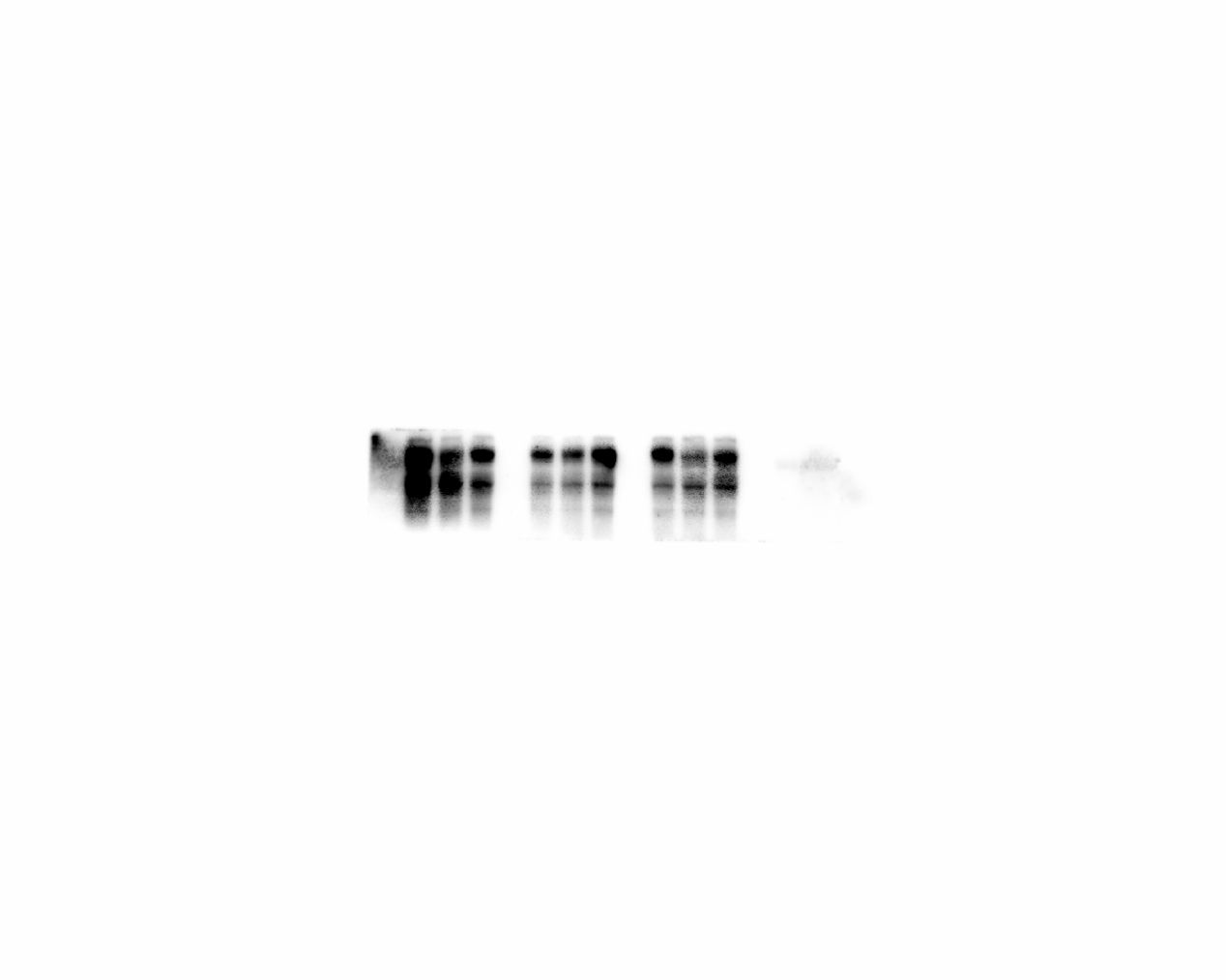
**

1. **Figure 2e, actin(1)**

**
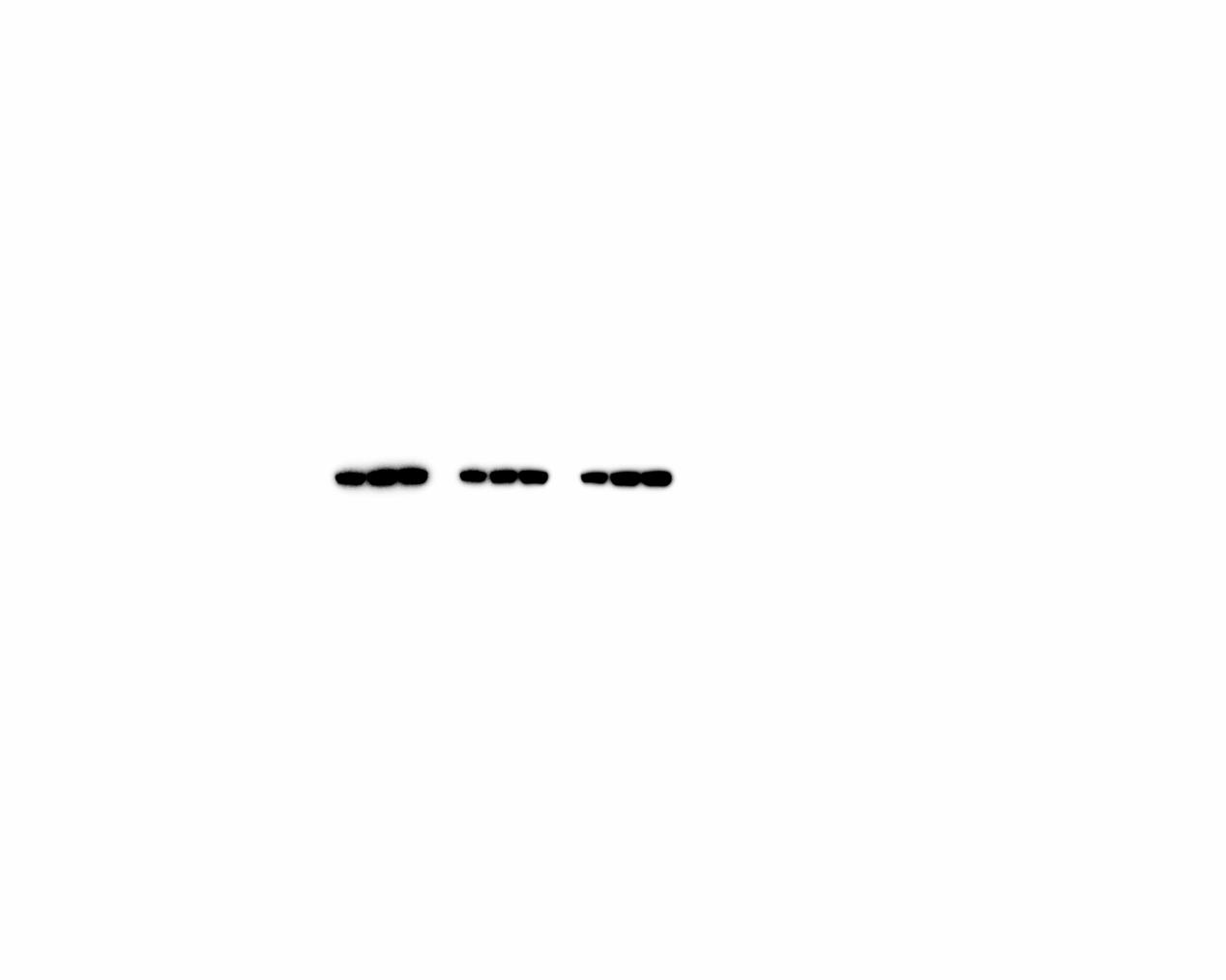
**

**Figure2e, actin(2)**

**
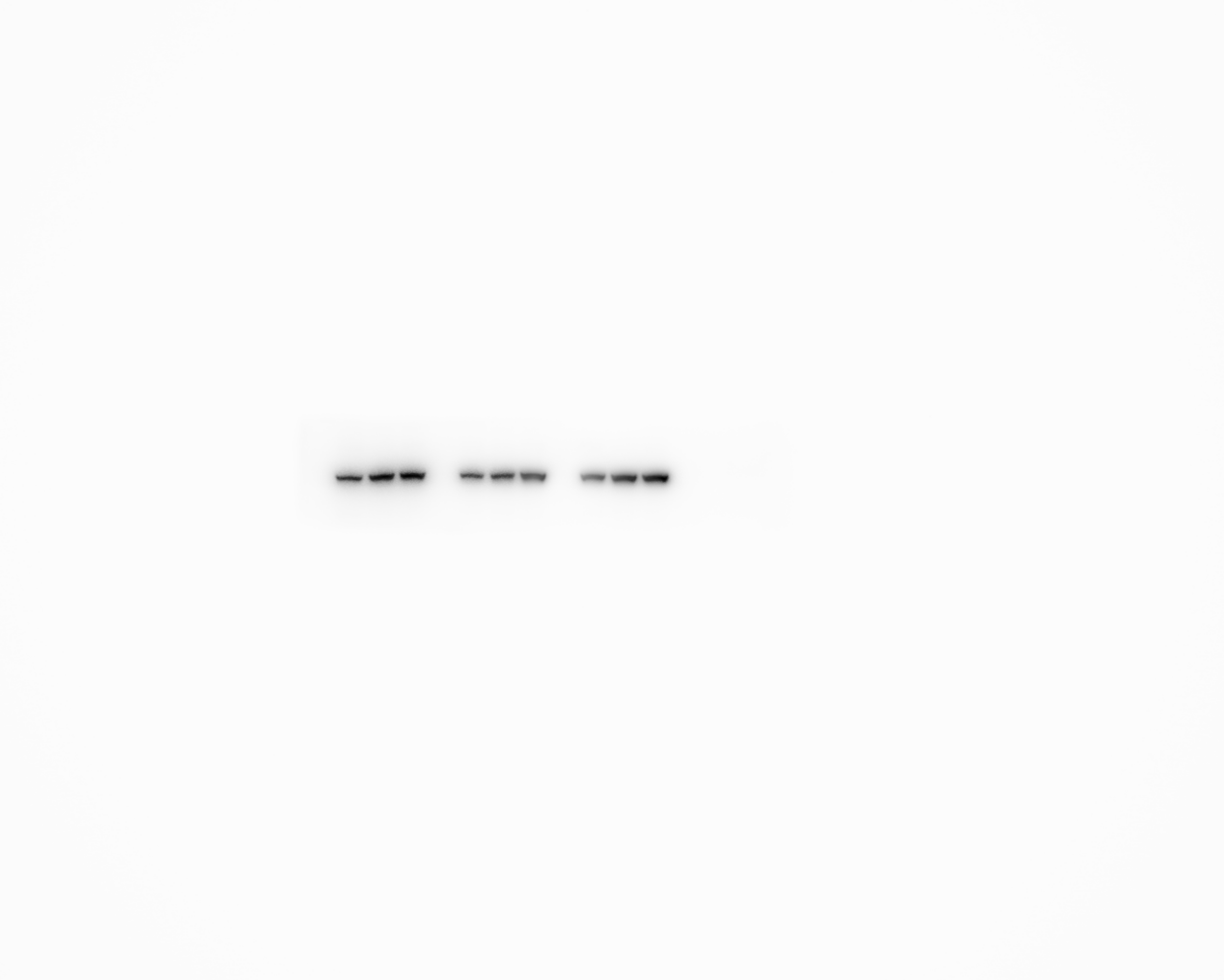
**

1. **Figure 3c, Per1(1)**

**
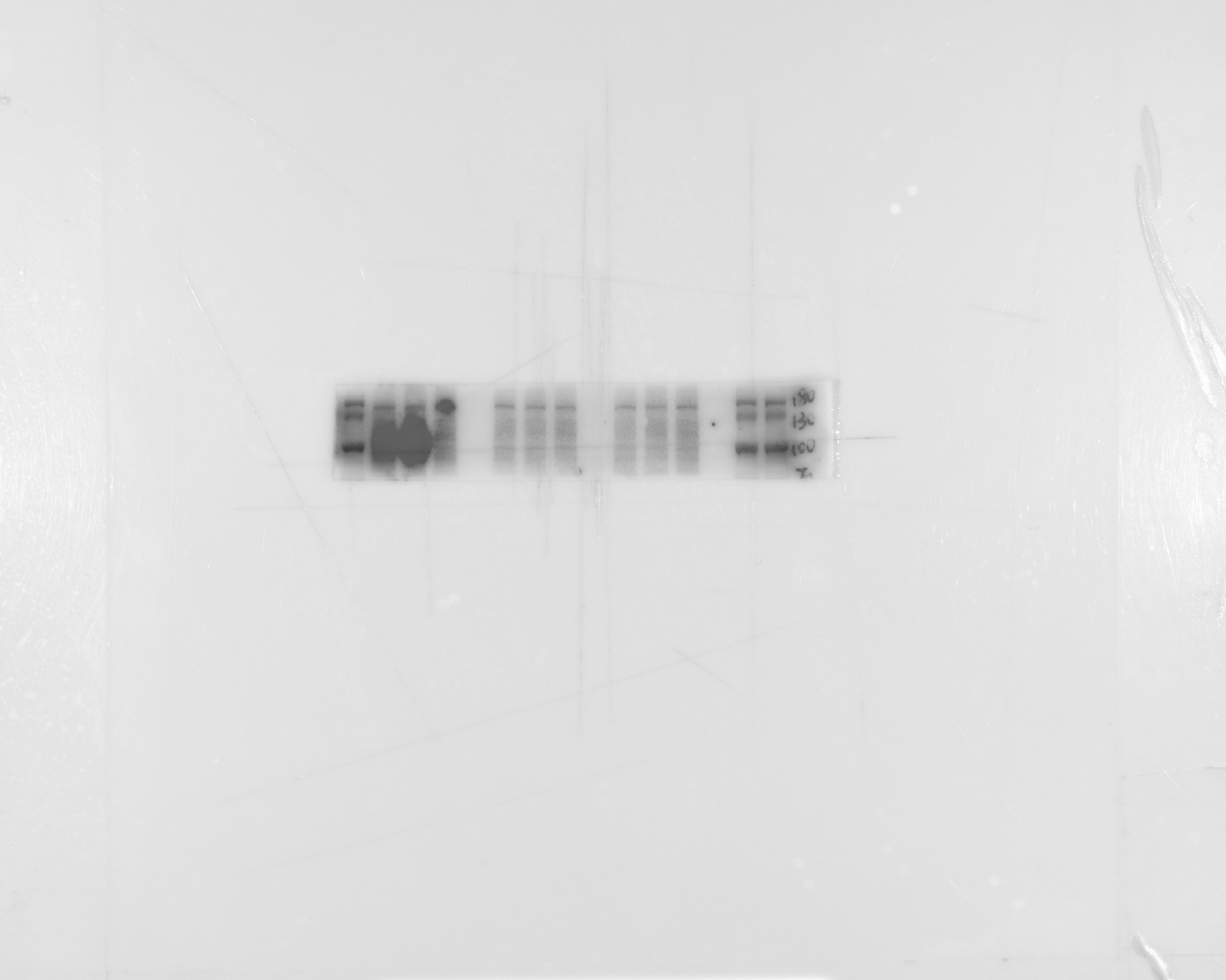
**

**Figure 3c, Per1(2)**

**
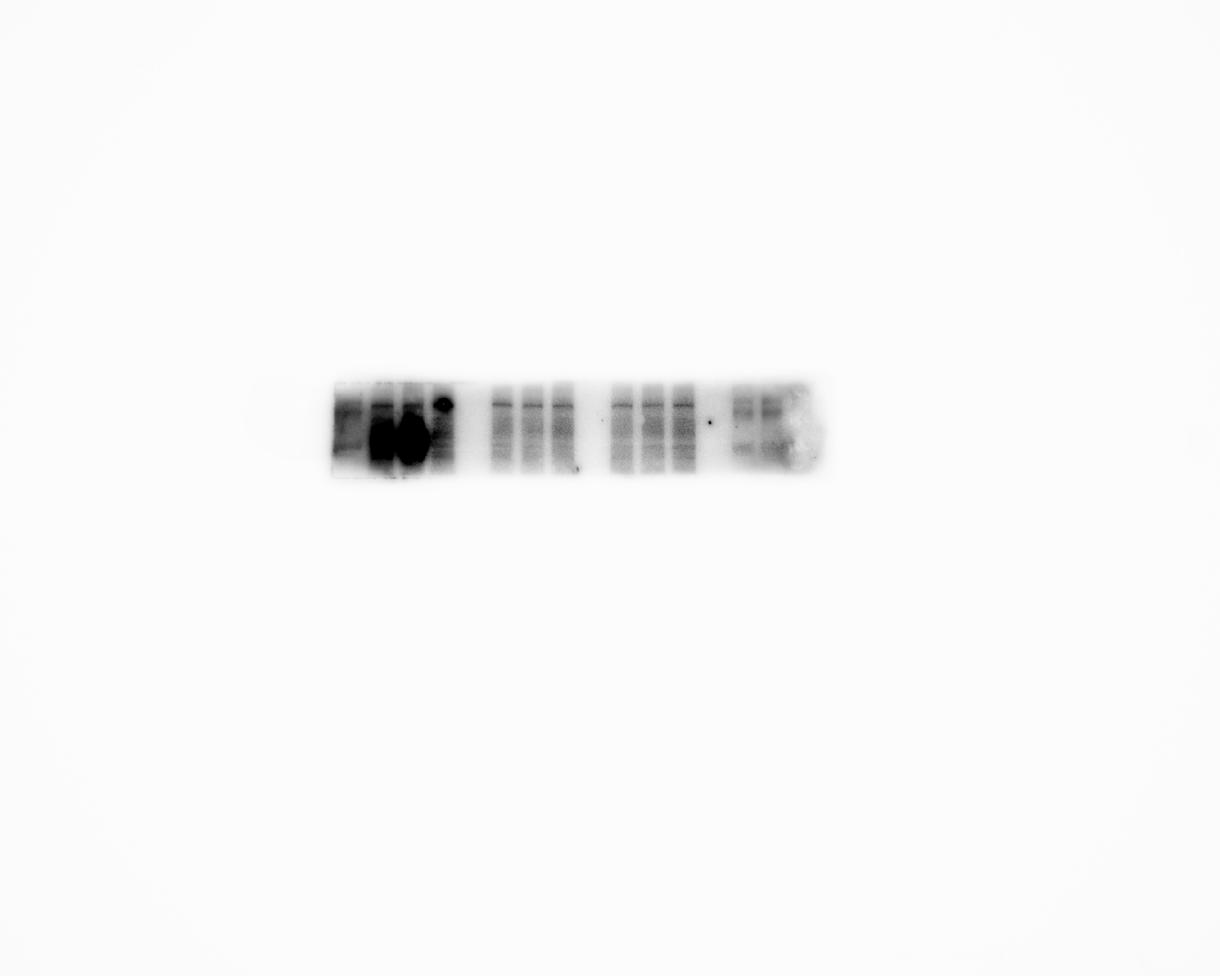
**

1. **Figure 3c, Gapdh**

**
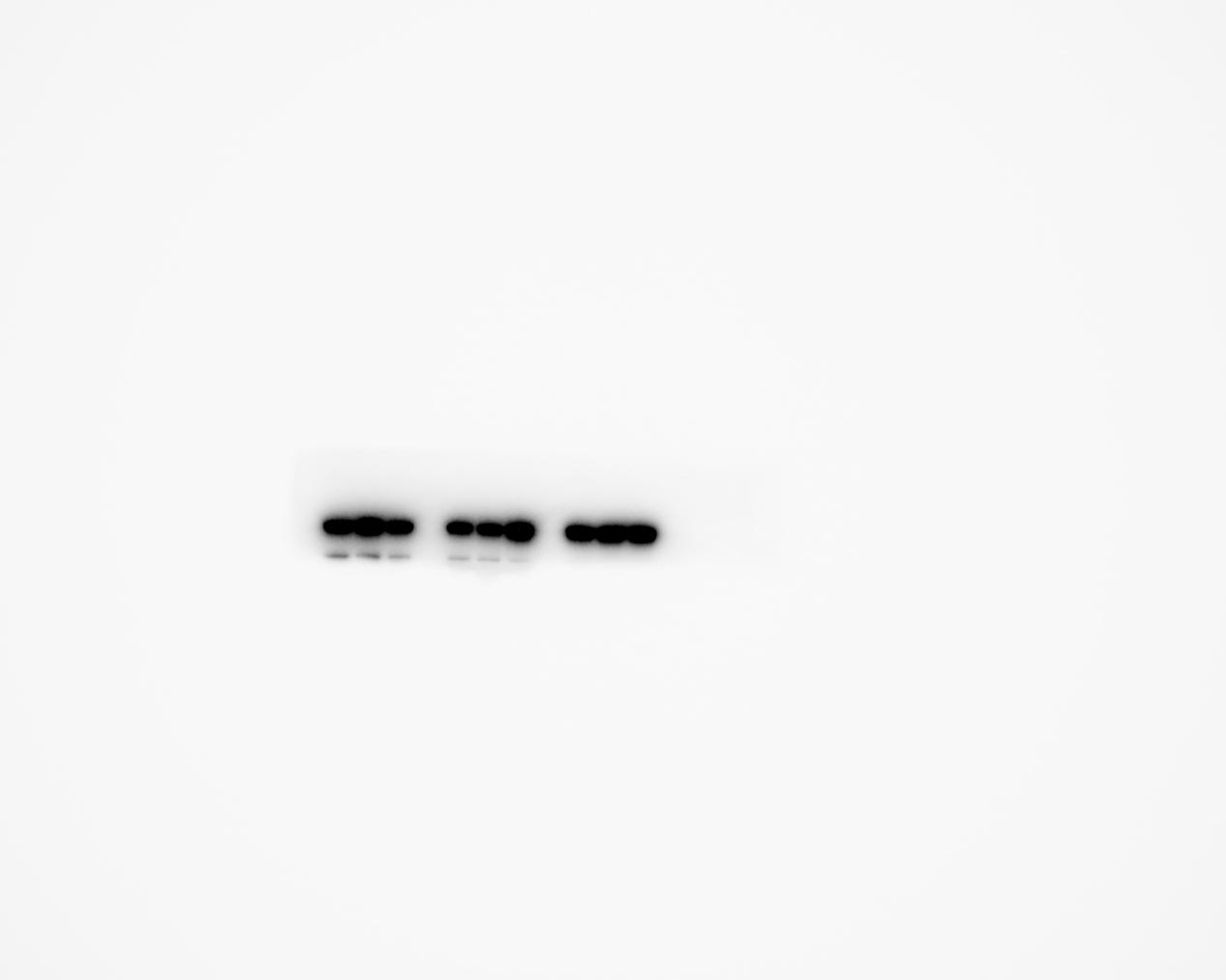
**

**Figure S1**

**IL17**

**
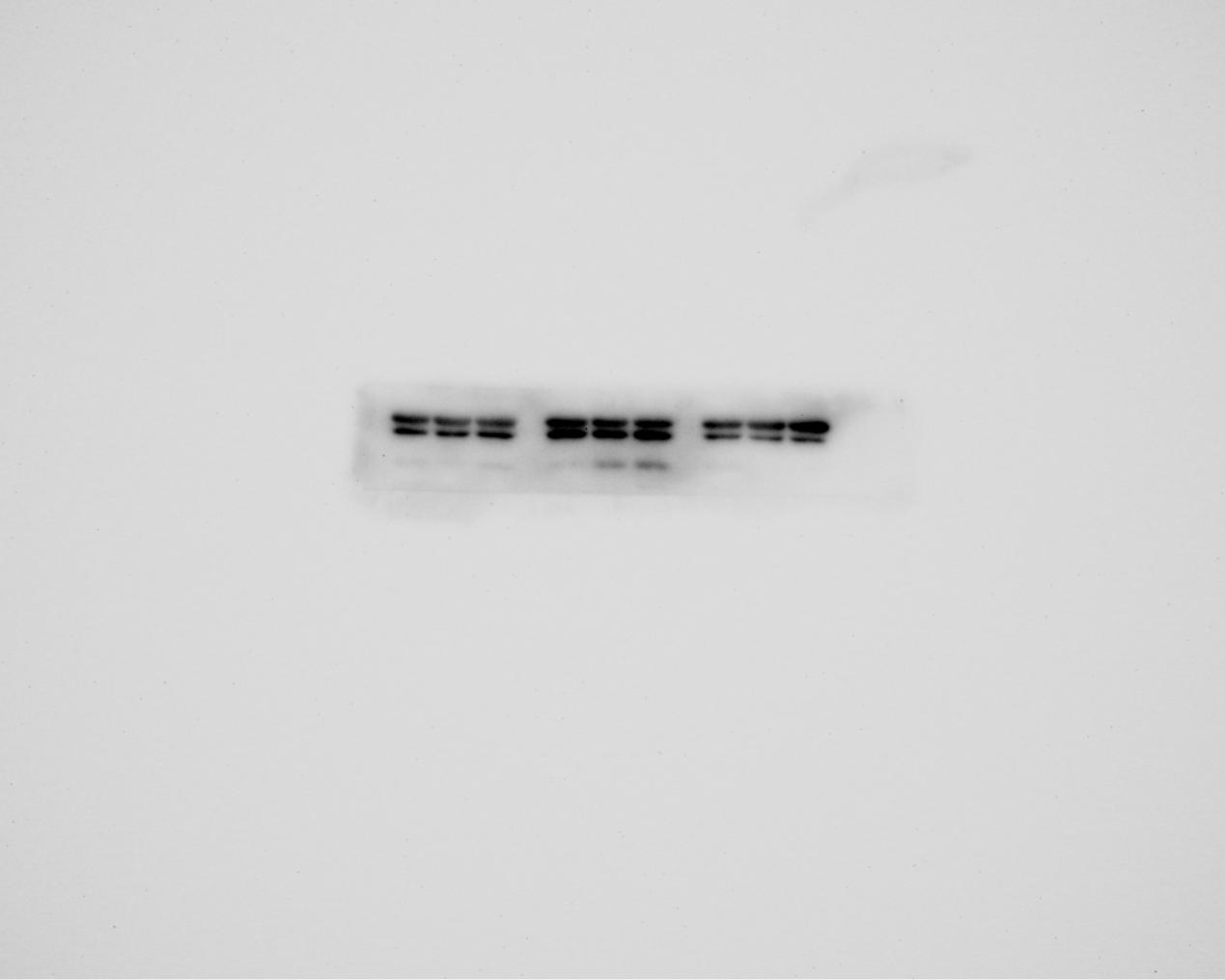
**

**Figure S1**

**Jak2**

**
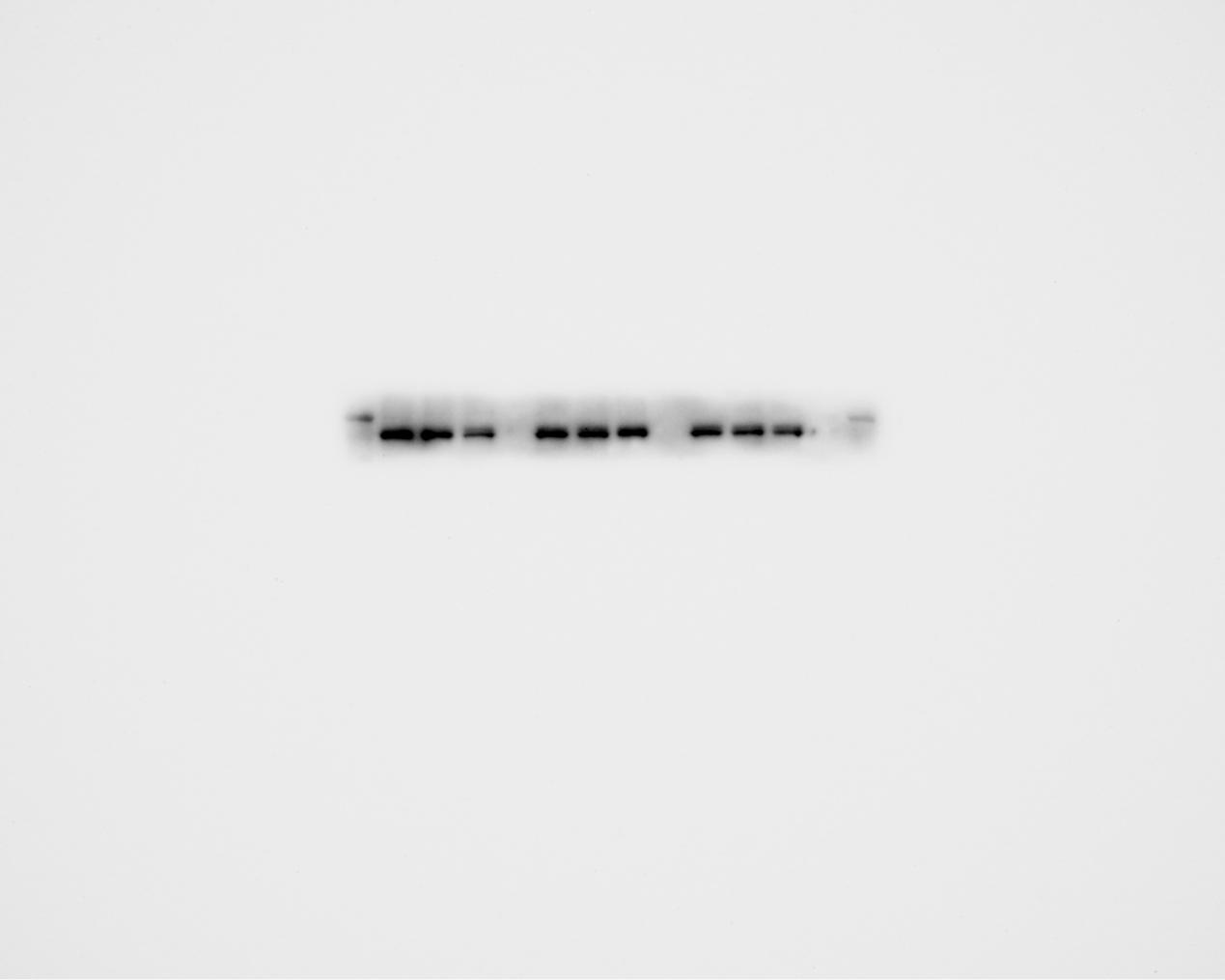
**

**Figure 1S**

**pJak2**

**
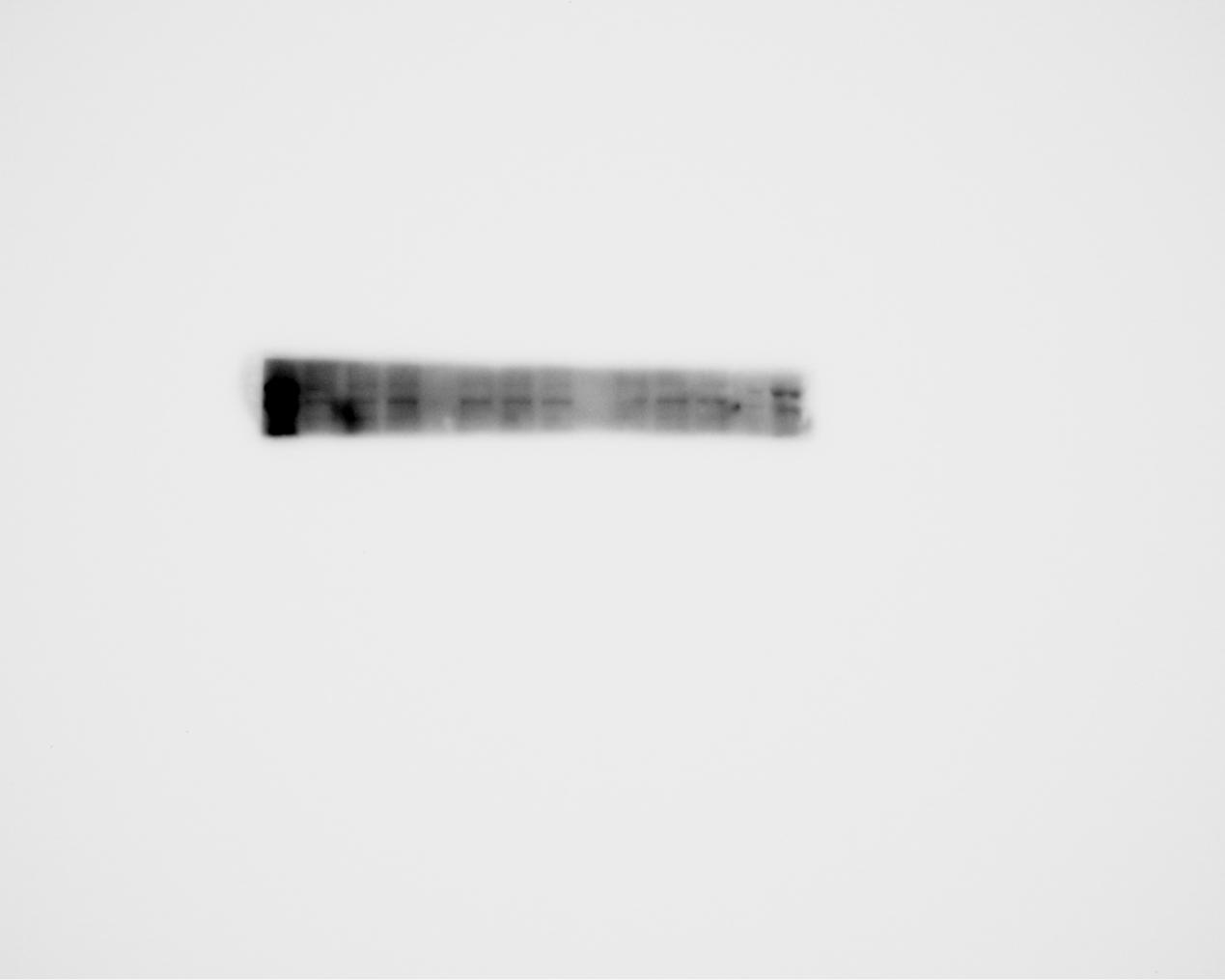
**

**Figure S1**

**Stat3**

**
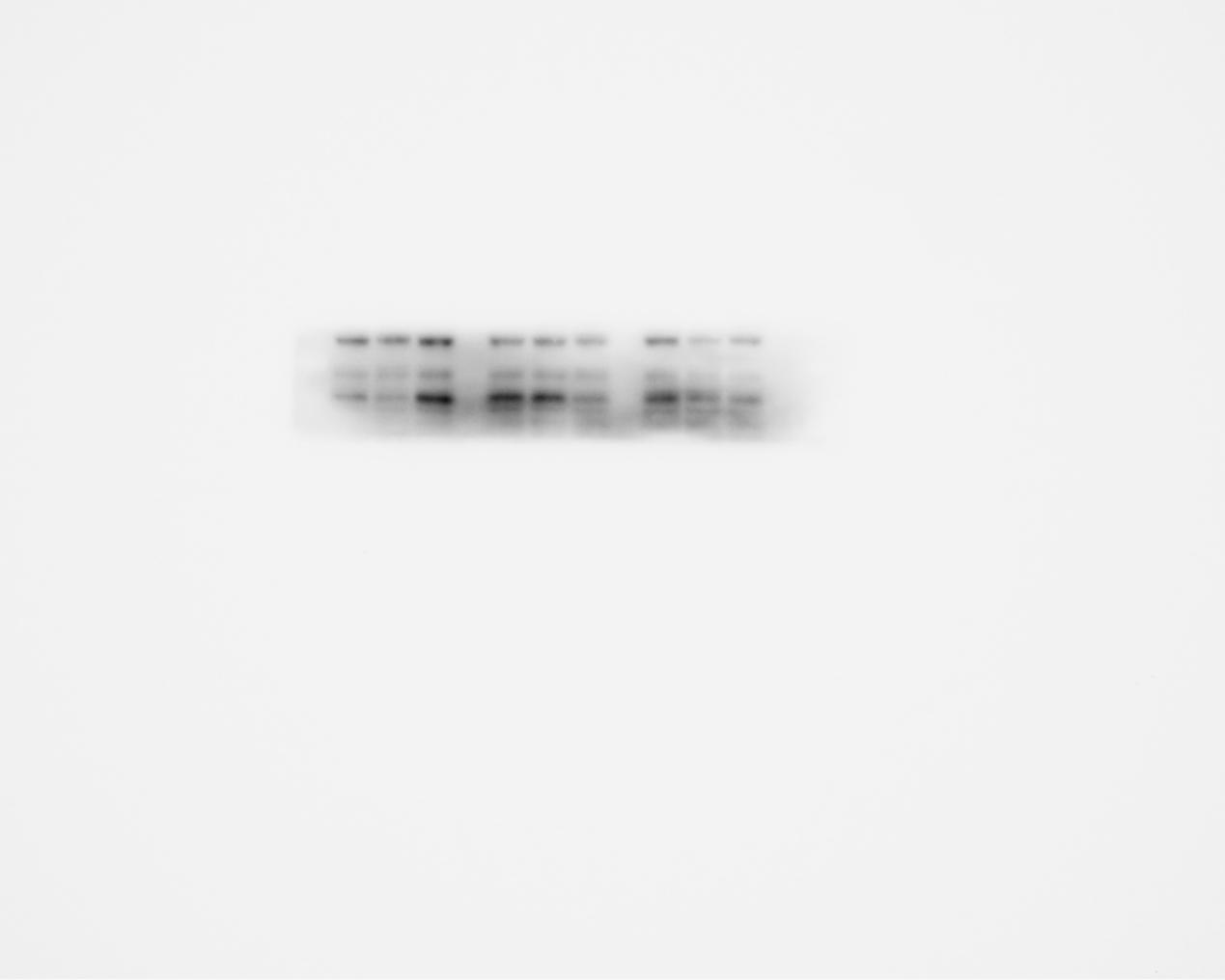
**

**Figure S1**

**pStat3**

**
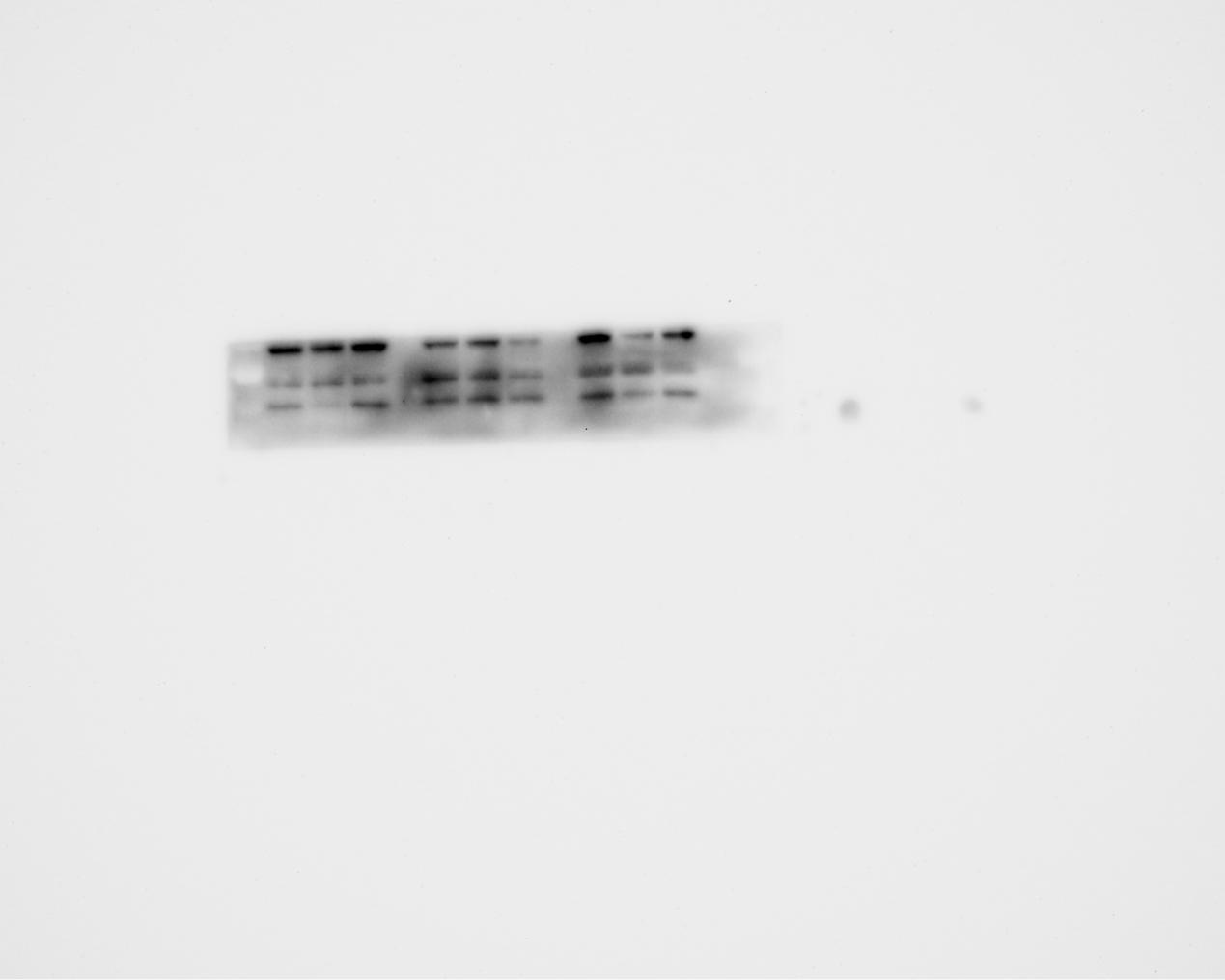
**

**Figure S1**

**Gapdh**

**
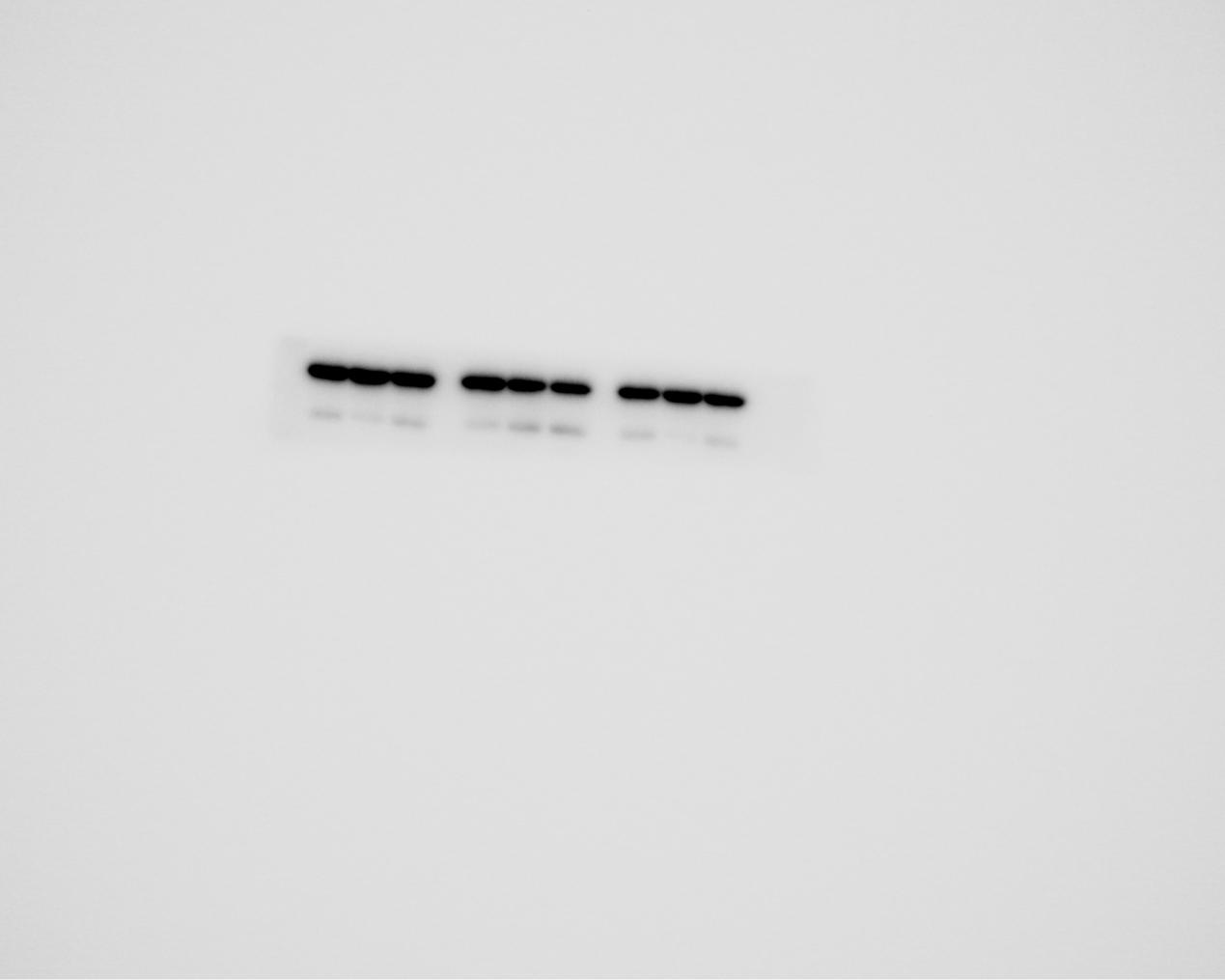
**
